# The complete mitochondrial genome of the endangered Assam Roofed Turtle, *Pangshura sylhetensis* (Testudines: Geoemydidae): Genomic features and phylogeny

**DOI:** 10.1371/journal.pone.0225233

**Published:** 2020-04-23

**Authors:** Shantanu Kundu, Vikas Kumar, Kaomud Tyagi, Kailash Chandra

**Affiliations:** Molecular Systematics Division, Centre for DNA Taxonomy, Zoological Survey of India, Kolkata, India; Imagine Institute, FRANCE

## Abstract

The Assam Roofed Turtle, *Pangshura sylhetensis* is an endangered and least studied species endemic to India and Bangladesh. The present study decodes the first complete mitochondrial genome of *P*. *sylhetensis* (16,568 bp) by using next-generation sequencing. The assembly encodes 13 protein-coding genes (PCGs), 22 transfer RNAs (tRNAs), two ribosomal RNAs (rRNAs), and one control region (CR). Most of the genes were encoded on the majority strand, except NADH dehydrogenase subunit 6 (*nad6*) and eight tRNAs. All PCGs start with an ATG initiation codon, except for Cytochrome oxidase subunit 1 (*cox1*) and NADH dehydrogenase subunit 5 (*nad5*), which both start with GTG codon. The study also found the typical cloverleaf secondary structures in most of the predicted tRNA structures, except for serine (*trnS1*) which lacks of conventional DHU arm and loop. Both Bayesian and maximum-likelihood phylogenetic inference using 13 concatenated PCGs demonstrated strong support for the monophyly of all 52 Testudines species within their respective families and revealed *Batagur trivittata* as the nearest neighbor of *P*. *sylhetensis*. The mitogenomic phylogeny with other amniotes is congruent with previous research, supporting the sister relationship of Testudines and Archosaurians (birds and crocodilians). Additionally, the mitochondrial Gene Order (GO) analysis indicated plesiomorphy with the typical vertebrate GO in most of the Testudines species.

## Introduction

The evolution of living organisms is a continuous process over generations and difficult to understand by measuring with a distinct speciation hypothesis [1]. Several biological as well as environmental factors play an important role in the mutations of a gene from one generation to the next, leading to an altered gene in a new species from an ancestral population. Apart from natural selection, the genetic traits of a population are often altered randomly, forced by several biotic/abiotic factors, gradually leading to the evolutionary dynamics of a species. It is evidenced that, the adequate gene sequences have been largely employed to elucidated the phylogeny and evolutionary patterns of earth's biota including reptiles [2,3]. Testudines (turtles, tortoises, and terrapins) are one of the oldest groups of living organisms on earth with an extended evolutionary history [4]. Besides using morphology, the genetic approach has been repeatedly applied to address the systematics of the group [5–7]. Both nuclear and mitochondrial genes have been extensively utilized for studying Testudines phylogeny and genetic diversity [8–10]. In particular, complete mitogenomes have been used to understand the deep evolutionary branching of the group, providing evidence of ‘Turtle-Archosaur affinity’ [11,12]. These results have been further complemented by phylogenomic analyses [13–15]. However, details of the internal phylogeny of Testudines still need to be reconciled and mining of large-scale complete mitochondrial genomes provide valuable data for this purpose.

Testudines mitochondrial genomes are circular and double stranded with a size of 16–19 kb and contain typically 37 genes [16–20]. Remarkably, some of the reptile species, including turtles, have variable numbers of genes in their mitogenome due to the loss of protein-coding genes (PCGs), duplication of control regions (CRs), and occurrence of multiple contiguous numbers of one or more transfer RNA genes (tRNAs) [21]. Besides the structural features of individual genes, the gene orders (GOs) in mitogenomes have proven useful for defining clades at different taxonomic levels in both vertebrates and invertebrates [22–26]. In general, the arrangements of GO are defined by transposition, inversion, inverse transposition, and tandem duplication and random loss (TDRL) mechanisms in comparisons with the typical ancestral GO [27]. Moreover, these evolutionary events also help to define the plesiomorphic/apomorphic status and transformational pathways of mitogenomes [28,29]. Thus, to infer the evolutionary pathways leading to the detected diversity of GOs, it is essential to test phylogeny and GO conjointly [30,31]. As of now, more than 100 mitogenomes of Testudines species of 56 genera within 12 families and two sub-orders have been generated worldwide to address several phylogenetic questions. However, a combined study on GO and phylogeny has never been performed for Testudines.

The evolutionarily distinct genus *Pangshura* is known from four extant species from Southeast Asian countries [32]. Among them, the Assam Roofed turtle, *Pangshura sylhetensis* is a highly threatened species and categorized as ‘Endangered’ in the International Union for Conservation of Nature (IUCN) Red data list [33]. The distribution of *P*. *sylhetensis* is restricted to Bangladesh and India [34,35]. Though some partial sequences of segments of the mitochondrial genome for this species are publicly available [32], the complete mitochondrial genome has not been sequenced. Therefore, the present study aimed to generate the complete mitochondrial genome of *P*. *sylhetensis* and execute a comparative analysis with other Testudines belonging to both sub-orders (Cryptodira and Pleurodira). Here, we used phylogenetic (Bayesian and Maximum Likelihood) and GO analyses to gain better insights into the Testudines evolutionary scenario.

## Materials and methods

### Ethics statement and sample collection

**Fig 1:**
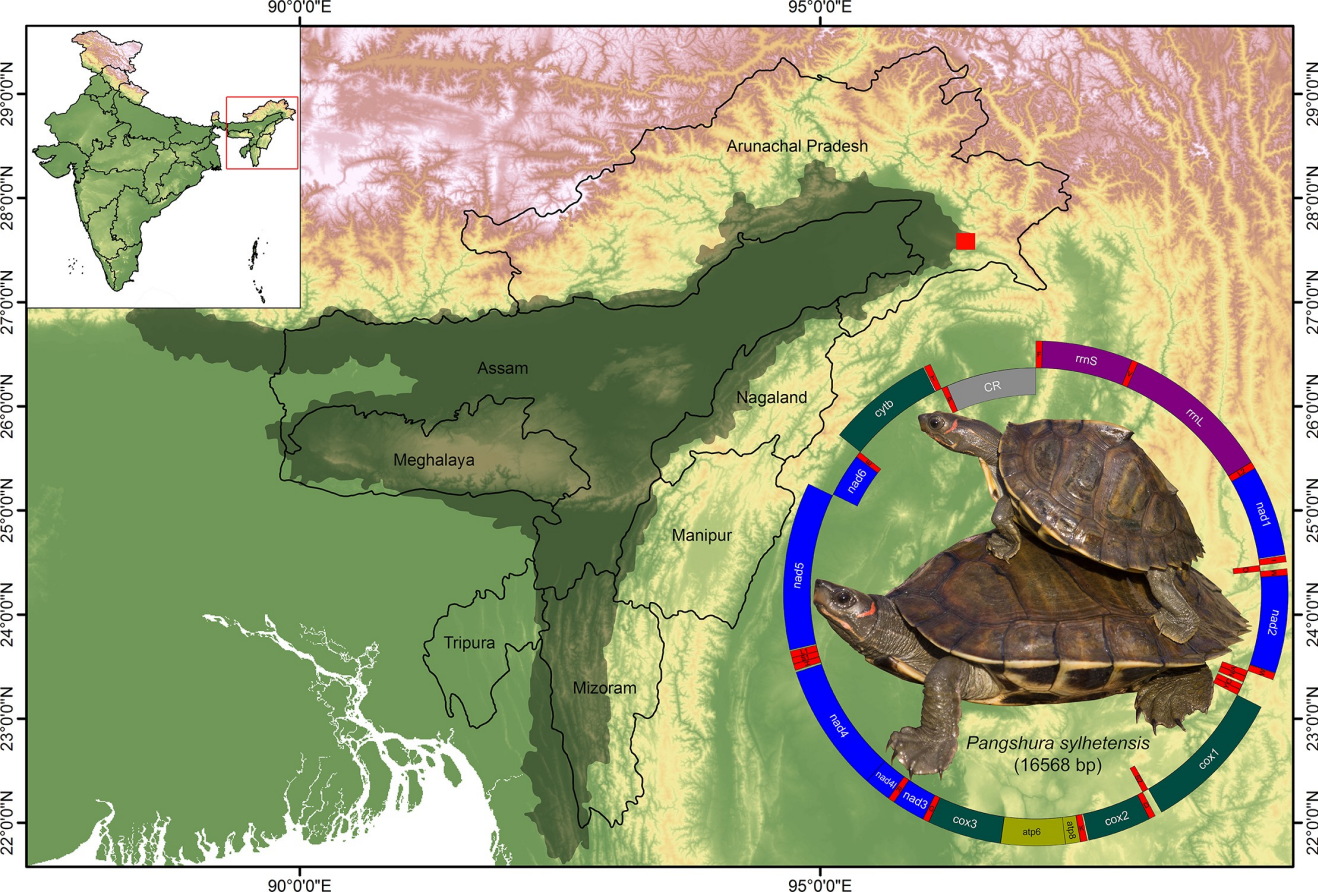
The spatial range distribution (marked by gray shadow) and collection locality (27.50 N 96.24 E marked by red square) of *P*. *sylhetensis*. The species photograph and mitochondrial genome of *P*. *sylhetensis*. Protein-coding genes are marked by blue, deep green, and yellowish-green color boxes, rRNA genes are marked by violet color boxes, tRNA genes are marked by red color boxes, and control region is marked by gray color box.

To conduct the field survey and biological sampling, prior consent was acquired from the wildlife officials of the state Arunachal Pradesh in northeast India (Letter No. SFRI/APBB/09‐2011‐1221‐1228 dated 22.07.2016). No turtle specimen was sacrificed in the present study. The *P*. *sylhetensis* specimen was collected from the tributaries of Brahmaputra River (Latitude 27.50 N and Longitude 96.24 E) from the nearby localities of Namdapha National Park in northeast India (Fig 1). The species photograph was taken by the first authors (S.K.) and edited manually in Adobe Photoshop CS 8.0. A blood sample (1 ml) was collected in sterile condition from the hind limb of the live individual by using micro‐syringe and stored in EDTA vial at 4°C. Subsequently the specimen was released back in the same eco-system with ample care and attention. The country level topology map was downloaded from the DIVA-GIS Spatial data platform (http://www.diva-gis.org/datadown) and overlaid by ArcGIS 10.6 software (ESRI®, CA, USA). The range distribution was marked manually in Adobe Photoshop CS 8.0 based on published records [36].

### Mitochondrial DNA extraction and sequencing

An aliquot of the collected blood sample (20 μl) was thoroughly blended with 1 ml working buffer (0.32 M Sucrose, 1 mM EDTA, 10 mM TrisHCl) and centrifuged at 700 × g for 5 min at 4°C to eliminate the nuclei and cell debris. The supernatant was collected in 1.5 ml Eppendorf tubes and centrifuged at 12,000 × g for 10 min at 4°C to precipitate the mitochondrial pellet. The pellet was re-suspended in 200 μl of buffer (50 mM TrisHCl, 25 mM of EDTA, 150 mM NaCl), with the addition of 20 μl of proteinase K (20 mg/ml) followed by incubation at 56°C for 1–2 hr and the mitochondrial DNA was extracted by Qiagen DNeasy Blood & Tissue Kit (QIAGEN Inc.). The DNA quality was visualized in 1% agarose gel electrophoresis, and the concentration was quantified by NANODROP 2000 spectrophotometer (Thermo Scientific).

### Mitogenome assembly and annotation

Complete mitogenome sequencing and assembly was carried out at Xcelris Labs Limited, Gujarat, India (http://www.xcelrisgenomics.com/). The genome library was sequenced using the Illumina platform (2x150bp PE chemistry) with an average library size of 545bp, to generate ~3.78 GB data (Illumina, Inc, USA). The total number of paired reads was 26,263,128. The raw reads were handled using the cutadapt tool (http://code.google.com/p/cutadapt/) for removing adapters and low-quality base trimming with a cutoff of the Phread quality score (Q score) of 20. The high-quality reads were down-sampled to 2 million reads using the Seqtk program (https://github.com/lh3/seqtk). High-quality paired-end data was assembled with NOVOPlasty v2.6.7 using default parameters [37]. The mitogenome of *Mauremys reevesii* (accession no. KJ700438) was used as a reference seed sequence for assembly, resulting in a 16568 bp (~16.5Kb) single contig. To validate the assembly obtained from the NOVOPlasty assembler, a similarity search was carried out in the GenBank database using BLASTn v2.2.28 (https://blast.ncbi.nlm.nih.gov). Further, the contig was subjected to confirmation by the MITOS v806 online webserver (http://mitos.bioinf.uni-leipzig.de). The DNA sequences of the protein coding genes (PCGs) were translated into putative amino acid sequences on the basis of the vertebrate mitochondrial genetic code. The exact initiation and termination codons were identified in ClustalX using other publicly available reference sequences of Testudines [38]. The mitogenome was submitted to the GenBank database (Accession No. MK580979) using the Sequin submission tool (S1 Fig).

### Data set construction and comparative analysis

The circular illustration of the generated mitogenome of *P*. *sylhetensis* was plotted using the CGView Server (http://stoth.ard.afns.ualbe.rta.ca/cgview_server/) with default parameters [39]. The strand direction and arrangements of each PCG, tRNA, and ribosomal RNA (rRNA) were also checked through the MITOS online server. The overlapping regions and intergenic spacers between the neighbor genes were counted manually through Microsoft Excel. The tRNA genes of *P*. *sylhetensis* were affirmed by the MITOS online server, tRNAscan‐SE Search Server 2.0 (http://lowel.ab.ucsc.edu/tRNAs.can-SE/) and ARWEN 1.2 [40,41]. The base composition of all stems (DHU, acceptor, TѱC, anticodon) was examined manually to discriminate Watson‐Crick, wobble, and uneven base pairing. On the basis of homology in the Refseq database (https://www.ncbi.nlm.nih.gov/refseq/), 51 Testudines mitogenomes representing nine families of Cryptodira and three families of Pleurodira were acquired from GenBank and integrated in the dataset for comparative analysis (S1 Table). The mitogenome size and nucleotide composition were calculated using MEGA6.0 [42]. The base composition skew was calculated as defined previously: AT skew = (A–T)/(A + T), GC skew = (G–C)/(G + C) [43]. The start and stop codons of each PCG were asserted through the Open Reading Frame Finder web tool (https://www.ncbi.nlm.nih.gov/orffinder/). To determine the location for replication of the L-strand and putative secondary structures, all Testudines CRs were analyzed through the online Mfold web server (http://unafold.rna.albany.edu). Due to the lack of proper annotation, the CRs of two species (*Amyda cartilaginea* and *Phrynops hilarii*) were missing and thus not incorporated in the comparative analysis.

### Phylogeny and Gene Order (GO) analyses

To assess the phylogenetic relationships of the Testudines mitogenomes, two datasets were prepared for analysis by Bayesian analysis (BA) and maximum‐likelihood (ML) methods. The first dataset includes all the PCGs of 52 Testudines mitogenomes including *P*. *sylhetensis* (S1 Table). As the targeted species is a Cryptodiran species, all the Pleurodiran species were treated as out-group taxa in the first dataset. Most of the previous studies used limited taxa to infer the phylogenetic position of Testudines relative to other amniotes. Hence, to fortify the existing hypothesis, 52 Testudines mitogenomes and 11 other amniotes mitogenomes (Whale: KU891394, Human: AP008580, Platypus: NC_000891, Opossum: AJ508398, Iguana: NC_002793, Lizards: AB080237, Snake: HM581978, Tuatara: AF534390, Bird: AP003580, Alligator: NC_001922, and Crocodile: HM488007) were incorporated in the second dataset (S1 Table). Among them, the database sequence of the sperm whale (*Physeter catodon*: KU891394) was used as out-group taxon. The PCGs were aligned individually by codons using the MAFFT algorithm in TranslatorX and the L‐INS‐i strategy with GBlocks parameters [44]. Finally, for each of the two datasets, the sequences of all PCGs were concatenated using SequenceMatrix v1.7.84537 [45]. As NADH dehydrogenase subunit 6 (*nad6*) is encoded on the light-strand [46–48], the reverse complement of *nad6* sequences along with other PCGs were used for phylogenetic tree construction. The suitable models for phylogenetic analyses were estimated by partitioning of each gene using PartitionFinder 2 [49] at the CIPRES Science Gateway V. 3.3 [50] (S2 Table). The BA tree was built through Mr. Bayes 3.1.2, and the MCMC was run for 100,000,000 generations with sampling at every 100^th^ generation, and 25% of samples were discarded as burn‐in [51]. The ML tree was constructed using the IQ‐Tree web server with 1000 bootstrap samples [52]. The BA and ML topologies for both datasets were further refined in iTOL v4 (https://itol.embl.de/login.cgi) for better visualization [53] and edited with Adobe Photoshop CS 8.0.

Further, to check the gene arrangement scenario, the most contemporary TreeREx analysis was adopted to infer the evolutionary pathways within the Testudines, leading to the observed diversity of the GOs. TreeREx can easily distinguish the putative GOs at the internal nodes of a reference tree as it works in a bottom-up manner through the iterative analysis of triplets or quadruplets of GOs to decide all the GOs in the entire tree [31]. In the TreeREx analysis, the most consistent nodes are considered to be most reliable and marked by green color, whereas nodes with the highest level of uncertainty are marked by red color. We used the default settings of TreeREx suggested on the website (http://pacosy.informatik.uni-leipzig.de/185-0-TreeREx.html) to analyze every node of the reference phylogenetic tree. To finalize the gene arrangements dataset of 52 Testudines (S3 Table), the insertion, deletion, and duplication of genes were reviewed as discussed in the previous studies [54–57].

## Results and discussion

### Mitogenome structure and organization

The mitogenome (16,568 bp) of the endangered Assam Roofed turtle, *P*. *sylhetensis* was determined in the present study (GenBank accession no. MK580979). The mitogenome contained 37 genes, comprising 13 PCGs, 22 tRNAs, 2 rRNAs, and a major non-coding CR. Among them, nine genes (*nad6* and 8 tRNAs) were located on the minority strand, while the remaining 28 genes were located on the majority strand (Table 1 and Fig 1). Across Testudines, the length of the mitogenome varied from 15,339 bp (*A*. *cartilaginea*) to 19,403 bp (*Stigmochelys pardalis*). Out of 52 Testudines species, 43 species showed strand symmetry as observed in typical vertebrates [58]. The gene arrangement among the Testudines species is discussed in more detail below. The nucleotide composition of the *P*. *sylhetensis* mitogenome was A+T biased (59.27%), as is the case in all other Testudines mitogenomes ranging from 57.76% (Pleurodiran species *P*. *hilarii*) to 64.19% (Cryptodiran species *Kinosternon leucostomum*) (S4 Table). The A+T composition of *P*. *sylhetensis* PCGs was 58.77%. The AT skew and GC skew were 0.124 and -0.334 in the mitogenome of *P*. *sylhetensis*. The comparative analysis showed that the AT skew ranged from 0.087 (*Testudo graeca*) to 0.208 (*Carettochelys insculpta*) and the GC skew from -0.296 (*S*. *pardalis*) to -0.412 (*Trionyx triunguis*) (S4 Table). A total of 15 overlapping regions with a total length of 73 bp were identified in *P*. *sylhetensis* mitogenome. The longest overlapping region (21 bp) was observed between tRNA‐Leucine (*trnL1*) and NADH dehydrogenase subunit 5 (*nad5*). Further, a total of 12 intergenic spacer regions with a total length of 129 bp were observed in *P*. *sylhetensis* mitogenome with a longest region (66 bp) between tRNA‐Proline (*trnP*) and CR (Table 1).

**Table 1:** List of annotated mitochondrial genes of *Pangshura sylhetensis*.

Gene	Direction	Location	Size (bp)	Anti- codon	Start codon	Stop codon	Intergenic Nucleotides
*trnF*	+	1–69	69	GAA	.	.	0
*rrnS*	+	70–1031	962	.	.	.	0
*trnV*	+	1032–1100	69	TAC	.	.	11
*rrnL*	+	1112–2697	1586	.	.	.	1
*trnL2*	+	2699–2774	76	TAA	.	.	0
*nad1*	+	2775–3743	969	.	ATG	TAG	-1
*trnI*	+	3743–3813	71	GAT	.	.	-1
*trnQ*	-	3813–3883	71	TTG	.	.	-1
*trnM*	+	3883–3951	69	CAT	.	.	0
*nad2*	+	3952–4992	1041	.	ATG	TAG	-2
*trnW*	+	4991–5064	74	TCA	.	.	-1
*trnA*	-	5064–5132	69	TGC	.	.	1
*trnN*	-	5134–5207	74	GTT	.	.	27
*trnC*	-	5235–5300	66	GCA	.	.	0
*trnY*	-	5301–5371	71	GTA	.	.	1
*cox1*	+	5373–6923	1551	.	GTG	AGG	-12
*trnS2*	-	6912–6982	71	TGA	.	.	0
*trnD*	+	6983–7052	70	GTC	.	.	0
*cox2*	+	7053–7739	687	.	ATG	TAG	1
*trnK*	+	7741–7813	73	TTT	.	.	1
*atp8*	+	7815–7982	168	.	ATG	TAA	-10
*atp6*	+	7973–8656	684	.	ATG	TAA	-1
*cox3*	+	8656–9440	785	.	ATG	TA(A)	-1
*trnG*	+	9440–9507	68	TCC	.	.	1
*nad3*	+	9509–9857	349	.	ATG	GAA	1
*trnR*	+	9859–9927	69	TCG	.	.	0
*nad4l*	+	9928–10224	297	.	ATG	TAA	-7
*nad4*	+	10218–11594	1377	.	ATG	TAA	14
*trnH*	+	11609–11677	69	GTG	.	.	0
*trnS1*	+	11678–11744	67	GCT	.	.	-1
*trnL1*	+	11744–11816	73	TAG	.	.	-21
*nad5*	+	11796–13628	1833	.	GTG	TAG	-8
*nad6*	-	13621–14145	525	.	ATG	AGG	-3
*trnE*	-	14143–14210	68	TTC	.	.	4
*cytb*	+	14215–15361	1147	.	ATG	T(AA)	-3
*trnT*	+	15359–15430	72	TGT	.	.	0
*trnP*	-	15431–15501	71	TGG	.	.	66
CR	.	15568–16389	822	.	.	.	.

### Protein‐coding genes

**Fig 2:**
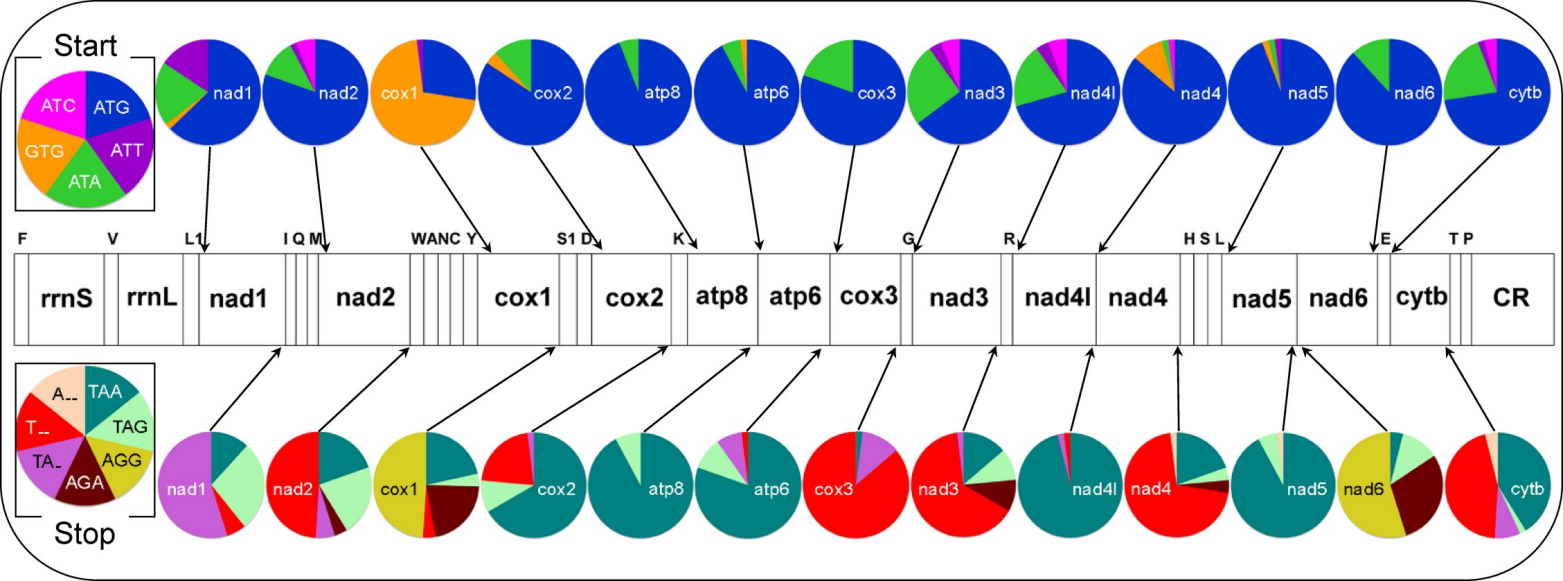
Frequency of start and stop codons for 13 protein-coding genes in the 52 Testudines mitogenomes.

The total length of PCGs was 11,268 bp in *P*. *sylhetensis*, which represents 68.01% of the complete mitogenome. The AT skew and GC skew were 0.056 and -0.345 in the PCGs of *P*. *sylhetensis* (S4 Table). Most of the PCGs of *P*. *sylhetensis* initiated with an ATG start codon; however, the GTG initiation codon was found in the Cytochrome oxidase subunit 1 (*cox1*) and *nad5* genes. The TAG termination codon was used by four PCGs, TAA by four PCGs, AGG by two PCGs, and GAA by one PCG. The incomplete termination codons TA(A) and T(AA) were detected in Cytochrome oxidase subunit 3 (*cox3*) and Cytochrome b (*cytb*) gene, respectively. The comparative analysis with other Testudines species revealed that the high frequency of initiation codons (ATN, GTG) was observed in NADH dehydrogenase subunit 1 (*nad1*), NADH dehydrogenase subunit 2 (*nad2*), NADH dehydrogenase subunit 3 (*nad3*), NADH dehydrogenase subunit 4L (*nad4L*), NADH dehydrogenase subunit 4 (*nad4*), *nad5*, and *cytb* genes. The ATG initiation codon is the most frequent in most of the PCGs (64.71% to 96.08%), except for *cox1* which preferentially contains a GTG start codon (72.55%). The complete termination codons are most frequent which was found in eight PCGs, however the incomplete termination codons are observed in five PCGs (S5 Table and Fig 2).

### Ribosomal RNA and transfer RNA genes

The total length of two rRNA genes of *P*. *sylhetensis* was 2,560 bp, compared to a range from 1,611 bp (*Caretta caretta*) to 2,685 bp (*Pelodiscus sinensis*) among other Testudines species in the present dataset. The AT content within rRNA genes was 58.98%, while the AT and GC skew were 0.272 and -0.173 respectively (S4 Table). A total of 22 tRNAs were found in the *P*. *sylhetensis* mitogenome with a total length of 1,550 bp. In other Testudines, the length of tRNAs varied from 1,407 bp (*A*. *cartilaginea*) to 1,684 bp (*Platysternon megacephalum*). The AT content within tRNA genes was 59.87%, while the AT and GC skew were 0.017 and 0.057, respectively (S4 Table). Among all the tRNA genes, 14 were found on the majority strand and eight tRNA genes (*trnQ*, *trnA*, *trnN*, *trnC*, *trnY*, *trnS2*, *trnE*, and *trnP*) on the minority strand. The present study also detected the anticodons of each tRNA gene. Most of the tRNA genes were predicted to be folded into classical cloverleaf structures, except *trnS1* (without Dihydrouridine (DHU) stem and loop (S2 Fig). The conventional base pairings (A = T and G≡C) were observed in most of the tRNAs [59]; however, wobble base pairing was observed in the stem of 13 tRNAs (*trnL2*, *trnN*, *trnA*, *trnW*, *trnP*, *trnE*, *trnR*, *trnG*, *trnC*, *trnY*, *trnS2*, *trnK*, and *trnQ*) (S2 Fig).

### Control regions

The CR of *P*. *sylhetensis* was typically distributed with three functional domains: the termination associated sequence (TAS), the central conserved (CD), and the conserved sequence block (CSB), as observed in other vertebrate CRs [60]. As compared to the TAS and CSB domain with varying numbers of tandem repeats, the CD domain consisted of highly conserved sequences. Hence, the pattern of CR was varied among different vertebrates, including Testudines [60,61]. The total length of *P*. *sylhetensis* CR was 1,067 bp, compared to a range of 600 bp (*Lepidochelys olivacea*) to 3,885 bp (*S*. *pardalis*) in other species in the present dataset. In the *P*. *sylhetensis* CR, the AT and GC skew was -0.025 and -0.249 (S4 Table). The TAS domain was ‘TACATA’, while the CSB domain was further divided into four regions: CSB-F (AGAGATAAGCAAC), CSB-1 (GACATA), CSB-2 (TTAAACCCCCCTACCCCCC), and CSB-3 (TCGTCAAACCCCTAAATCC). The CR is also involved in the initiation of replication and is positioned between *trnP* and *trnF* for most of the Testudines except *P*. *megacephalum* [54,56]. In *P*. *sylhetensis*, 27 bp are present between CSB-1 and the stem-loop structure; however, in other species, this distance ranged from zero to 90 bp (*T*. *triunguis*) (S3 and S4 Figs). Overall, the structural features of the replication of the L-strand and putative secondary structures are species-specific in most of the Testudines species and could be used as a species-specific marker.

### The major phylogenetic relationship of Testudines

**Fig 3:**
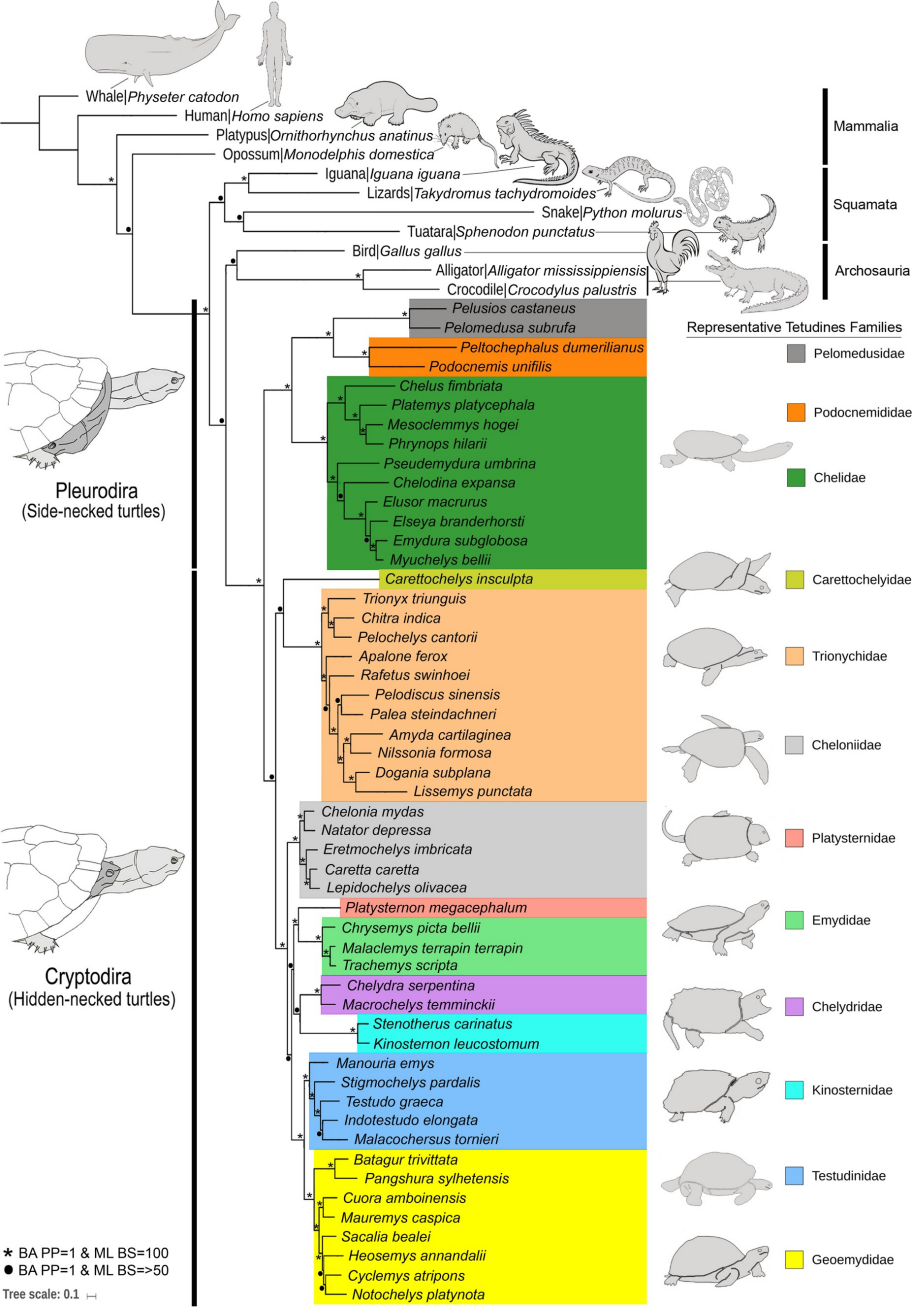
Unified Bayesian (BA) phylogenetic tree based on the concatenated nucleotide sequences of 13 PCGs of 52 Testudines species mitogenomes and 11 other amniotes showing the evolutionary relationship. Color boxes indicate the family level clustering for the studied Testudines species. The BA posterior probability support and ML bootstrap support of each node were superimposed. The nodes with high support values (posterior probability = 1 and bootstrap support = 100) were marked by asterisks. The nodes with high BA posterior probability support (1) and moderate/low ML bootstrap support (>50) were marked by black dot. Representative organism line diagrams were acquired from the internet.

The phylogenetic position of Testudines in the Vertebrate tree of life has been repeatedly evaluated in the last few decades [10–15]. Several approaches have been aimed at reconciling their phylogeny to obtain a better understanding of their origin and diversification [4]. Besides morphological parameters, the gene-based topology have been widely used for species identification, delimitation, and population genetics studies [62,63]. Both morphological and molecular data corroborated to erect all the *Pangshura* species from the closest congeners of *Batagur* [32,64,65]. Mitogenomic data has been successfully used to infer the phylogenetic relationships of many Testudines species, including the studied genus *Pangshura* within the Geoemydidae [66]. Already, the majority of species of the sub-family Geoemydinae have had their mitochondrial genome sequenced throughout the world. Nevertheless, the mitogenomes of only two species of sub-family Batagurinae are available in the global database. Hence, the present study adds the complete mitochondrial genome of the third Batagurinae species (*P*. *sylhetensis*). Both BA and ML methods inferred similar phylogenies and effectively discriminated all the studied Testudines species compiled in the first dataset with high posterior probability and bootstrap support (Fig 3, S5 and S6 Figs). The present phylogeny supports the sister relationship of *P*. *sylhetensis* with *Batagur trivittata* as described in previous phylogenies [66]. The other representative Testudines species also show strongly supported clustering within their respective families and sub-orders, consistent with previous phylogenetic hypotheses [63,65]. The present mitogenomic phylogeny with concatenated of 13 PCGs was adequate to infer a robust phylogeny and illuminate the relationship between *P*. *sylhetensis* and other Testudines species. We suggest that the collection of additional mitogenomic information of more taxa from different taxonomic lineages and diverse localities would be worthwhile to comprehensively elucidate the phylogeny of Testudines.

The phylogenetic position of Testudines has been a subject of research for a long time, with early studies resulting in controversial and inconclusive findings [67–69]. To resolve the phylogenetic placement of Testudines either among anapsids or diapsids, the mitogenome of Pleurodiran species has been analyzed resulting in the rejection of the placement of Testudines in the basal position in the amniote tree of life [11,70]. The mitogenome sequences have also been studied to evaluate the relationships between Archosaurians (birds and crocodilians) and Lepidosaurians (tuatara, snakes, and lizards) [71,72]. In the recent past, a reptilian transcriptome based phylogenetic analysis also suggests that, Testudines are not basal to extant reptiles but show close affinity towards other Archosaurians [73]. Further, candidate nuclear protein-coding locus (NPCL) markers were also evaluated, and turtles were robustly recovered as the sister group of Archosauria (birds and crocodilians), with an inferred evolutionary timescale congruent with the TimeTree of Life [74,75]. Furthermore, phylogenomic and phylotranscriptomic approaches as well as ultra-conserved elements (UCEs)-based consolidation were endeavoring to test the prevailing hypothesis and supported the sister relationships of Testudines with Archosaurians [13,76–78]. To further test the preferred phylogenetic hypothesis, we constructed the phylogenetic tree based on Testudines mitogenome dataset (52 Testudines + 11 other amniotes). In the phylogeny proposed by BA, the Archosaurians (birds, alligator, and crocodile) were clustered together (with posterior probability support 0.9) and showed a sister relationship with the Testudines (with posterior probability support 1) (Fig 3). In the phylogeny preferred by ML analysis, birds (*Gallus gallus*) was found as sister taxon of Testudines (with low bootstrap support 61), and the Alligator (*Alligator mississippiensis*)/Crocodile (*Crocodylus palustris*) lineage was recovered as sister to the birds/Testudines lineage (with moderate bootstrap support 72) (S6 Fig). We suspected the phylogenetic ambiguities were the result of biased sites across the mitochondrial PCGs that have a high chance of comprising biased signal for a particular phylogenetic relationship [13]. Nevertheless, the ML tree also supported the monophyly of the clade Archosauria + Testudines. Thus, although the BA and ML phylogenies showed shallow discrepancies in their branching patterns, the present mitogenome-based analyses are congruent with the prevailing hypothesis (diapsid affinity and sister relationship with Archosaurians) in comparison with other amniotes [11,13].

### Gene arrangements

**Fig 4:**
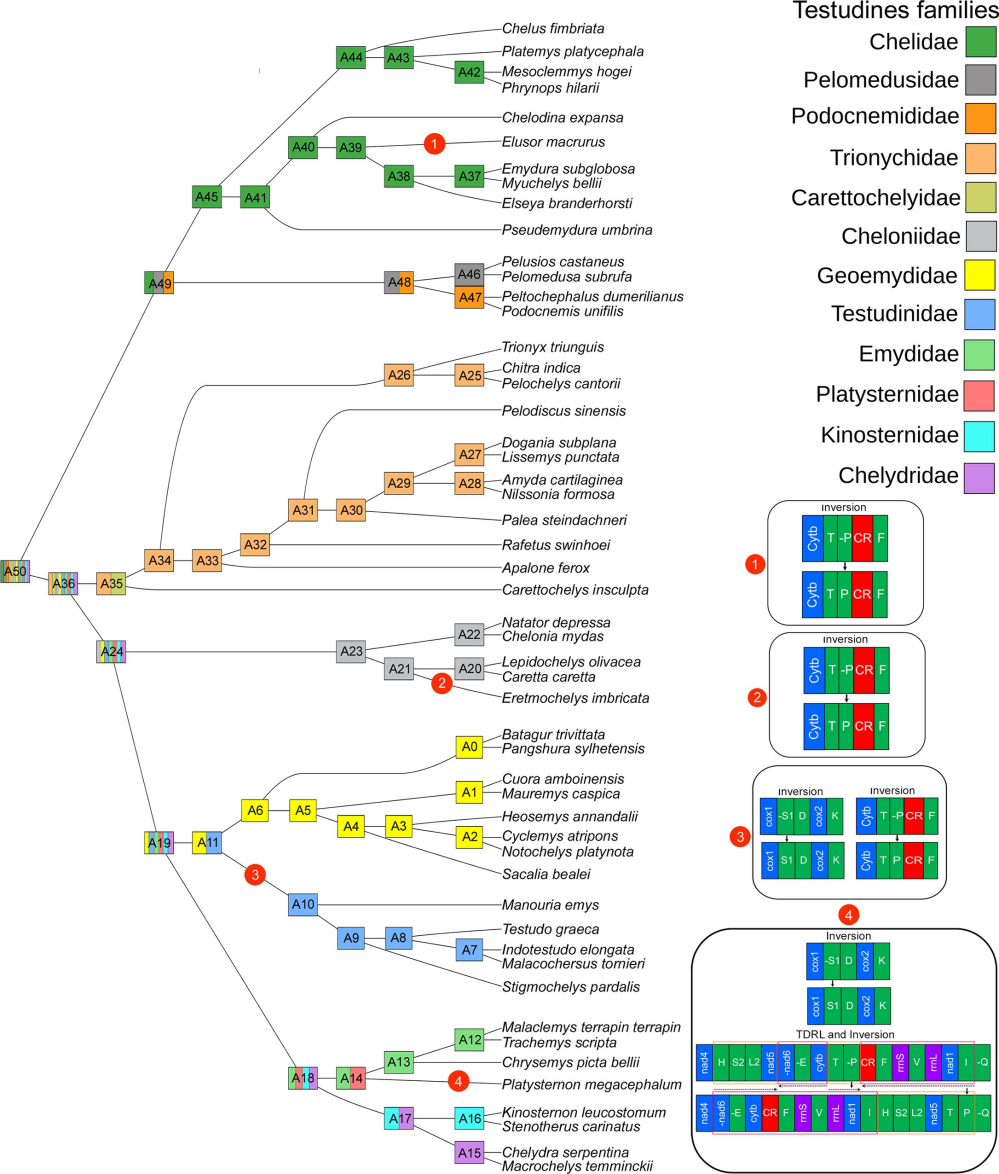
Gene-order based topology based on TreeREx analysis revealed the gene arrangement scenario within 52 Testudines species. All internal nodes are shared color as per phylogenetic clustering. Four gene arrangement scenarios are superimposed next to the tree.

To infer the evolutionary pathways among Testudines, TreeRex analysis was adopted to analyze the mitogenome gene arrangements within the order. A total of 50 consistent nodes were detected in the present analysis (Fig 4). Considering the A50 node as an ancestral trait of both Cryptodiran and Pleurodiran species in the present dataset, the gene arrangements are plesiomorphic for most of the Testudines species with few exceptions. Four gene rearrangements events were detected in the present dataset: (i) an inversion of *trnP* was observed in A39 node of *E*. *macrurus*, (ii) an inversion of trnP was observed in A21 node of *E*. *imbricata*, (iii) two inversions of *trnP* and *trnS1* were observed on the node A11 towards A10 which separates the family Testudinidae from Geoemydidae, (iv) two inversion of *trnS1*, *trnP* and one TDRL event towards *P*. *megacephalum* separate the family Platysternidae to Emydidae (Fig 4). However, the presumed synapomorphy was observed in three species robustly placed in three different families, Chelidae (*E*. *macrurus*), Cheloniidae (*E*. *imbricata*), and Platysternidae (*P*. *megacephalum*). As compared with the gene arrangement of other species within Chelidae and Cheloniidae, the GO of both *E*. *macrurus* and *E*. *imbricata* might be the result of parallel evolution, which needs further investigation. Furthermore, to clarify the phylogenetic position and evolutionary history of the sole member (*P*. *megacephalum*) of the monotypic family Platysternidae, the mitogenome data provide interesting new insights. An unusual gene arrangement, duplication of CR, and loss of redundant genes was observed in the *P*. *megacephalum* mitogenome [54,55,57]. Moreover, a micro-evolutionary analysis has shown that, the duplicate CR in *P*. *megacephalum* is derived from a heterologous ancestral recombination of mitochondrial DNA [56]. The present TreeRex-based GO analysis revealed that both inversion and TDRL events play a major role in the independent evolution of *P*. *megacephalum* as observed in earlier studies. The recently sequenced draft genome of this species will provide further clarification of its phylogenetic position [79].

## Conclusions

The evolutionarily distinct and globally endangered (EDGE) freshwater turtle, *P*. *sylhetensis* (family Geoemydidae), is endemic to India and Bangladesh. Due to habitat fragmentation, anthropogenic threats and illegal poaching, the populations of *P*. *sylhetensis* have remarkably declined across its range [80–82]. Hence, *P*. *sylhetensis* is categorized as an ‘endangered’ species in the IUCN Red data list, ‘Appendix II’ in the Convention on International Trade in Endangered Species of Wild Fauna and Flora (CITES), and ‘Schedule I’ species in Indian Wildlife (Protection) Act, 1972. The present study assembled and characterized the first complete mitogenome of *P*. *sylhetensis* (16,568 bp) and placed it in the context of a comparative analysis with other 52 Testudines representing 12 families and two sub-orders (Cryptodira and Pleurodira). The sequence features of the generated mitogenome and comparative analysis with other Testudines elucidate the structural variation of the mitochondrial molecules. The estimated mitogenome phylogenies indicate that *P*. *sylhetensis* is closely related to *B*. *trivittata*, consistent with previous data [66]. The present phylogenetic analyses (BA and ML) considering a larger set of mitogenomes support previous findings on diapsid affinity and the sister relationship of Testudines with Archosaurians. We suggest that the generation of high-throughput sequence information is required across Amniota lineages (including Testudines) to improve the understanding of their in-depth phylogenetic and evolutionary relationships. The GO analysis also revealed that most Testudines species show similar mitogenome gene arrangements as observed in typical vertebrates with few exceptions (*E*. *macrurus*, *E*. *imbricata*, *P*. *megacephalum*, and shared nodes of Geoemydidae-Testudinidae).

## Supporting information

S1 Fig A pictorial overview of the methodologies used for sequencing and analysis of *P*. *sylhetensis* mitogenome and bioanalyzer profiles after sonication of enriched mitochondrial DNA sample and libraries. (TIF)

S2 Fig Putative secondary structures for 22 tRNA genes in mitochondrial genome of *P*. *sylhetensis*. The first structure shows the nucleotide positions and details of stem-loop of tRNAs. The tRNAs are represented by full names and IUPAC-IUB single letter amino acid codes. Different base pairings are marked by red, blue and green color bars respectively.
(TIF)

S3 Fig Comparison of control region (CR) stem-loop structures of the origin of L-strand replication of 24 Testudines mitochondrial genomes. (TIF)

S4 Fig Comparison of control region (CR) stem-loop structures of the origin of L-strand replication of 26 Testudines mitochondrial genomes. (TIF)

S5 Fig Maximum Likelihood (ML) phylogenetic tree based on the concatenated nucleotide sequences of 13 PCGs of 52 Testudines species. Color boxes indicate the family level clustering for the studied species. The ML tree is drawn by IQ-Tree with bootstrap support values were indicated along with each node.
(TIF)

S6 Fig Maximum Likelihood (ML) phylogenetic tree based on the concatenated 13 PCGs of Testudines and other amniotes mitochondrial genomes. Color boxes indicate the family level clustering for the studied Testudines species. The ML tree is drawn by IQ-Tree with bootstrap support values were indicated along with each node.
(TIF)

S1 Table List of mitogenome sequences of Testudines and other amniotes species acquired from the NCBI database. (DOC)

S2 Table Estimated models by partitioning the 13 PCGs separately through PartitionFinder 2 for phylogenetic analyses of two datasets (52 mitogenomes of Testudines and 63 mitogenomes of Testudines + other amniotes). (DOC)

S3 Table Gene arrangements of the studied Testudines species used in the TreeREx analysis. (DOC)

S4 Table Nucleotide composition of 52 Testudines species mitochondrial genomes. The A+T biases of whole mitogenome, PCGs, tRNAs, rRNAs, and CRs were calculated by AT-skew = (A-T)/(A+T) and GC-skew = (G-C)/(G+C), respectively.
(DOC)

S5 Table Frequency of start and stop codon distribution within the complete mitogenomes of 52 Testudines. (DOC)
