# Supplementary figures and images for: The complete mitochondrial genome of the endangered Assam Roofed Turtle, Pangshura sylhetensis (Testudines: Geoemydidae): Genomic features and phylogeny
Source: PLoS One. 2020 Apr 23;15(4):e0225233. doi: 10.1371/journal.pone.0225233 (PMC7179895; doi:10.1371/journal.pone.0225233)

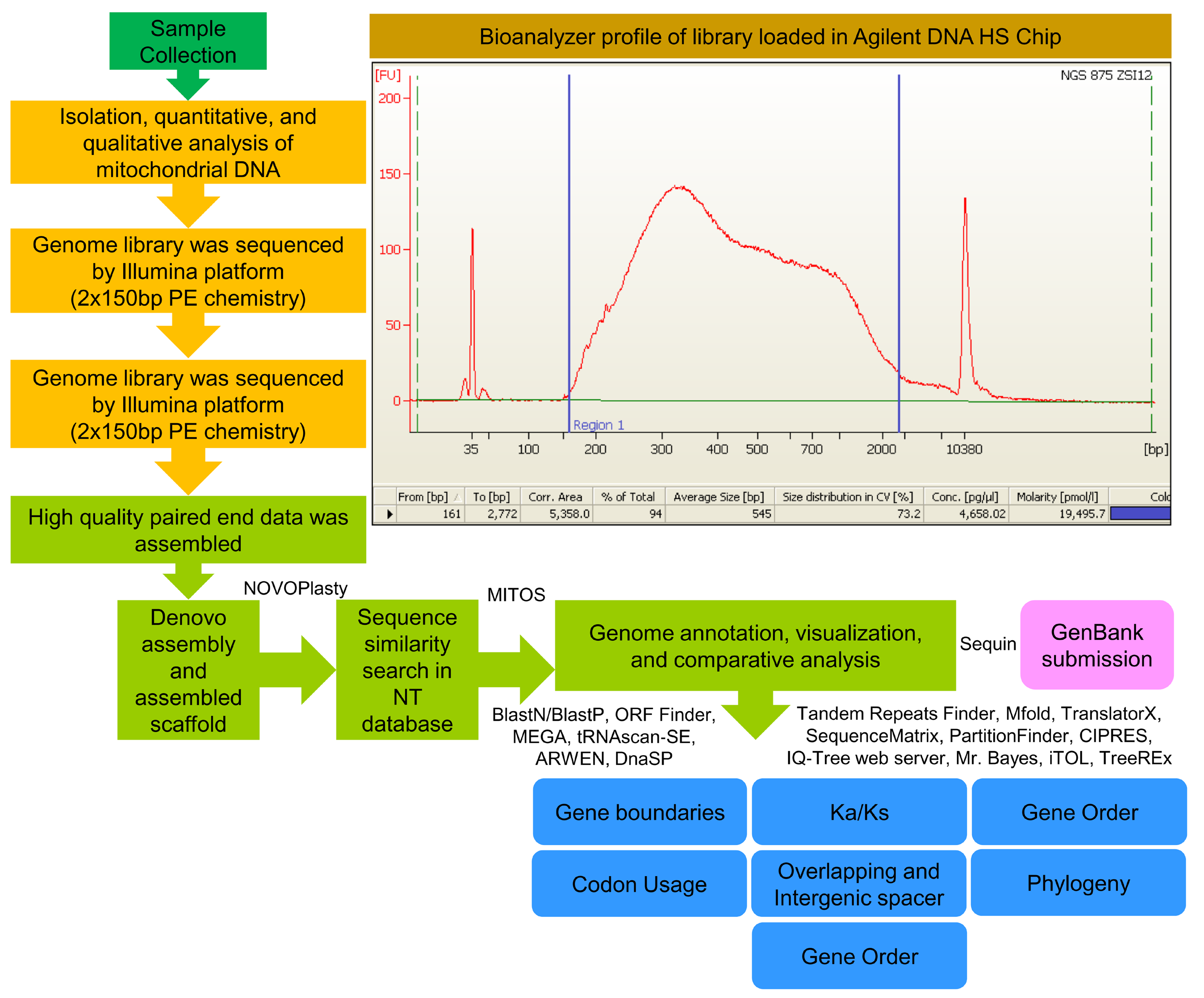

Supplement: S1 Fig — (TIF) [file pone.0225233.s001.tif]

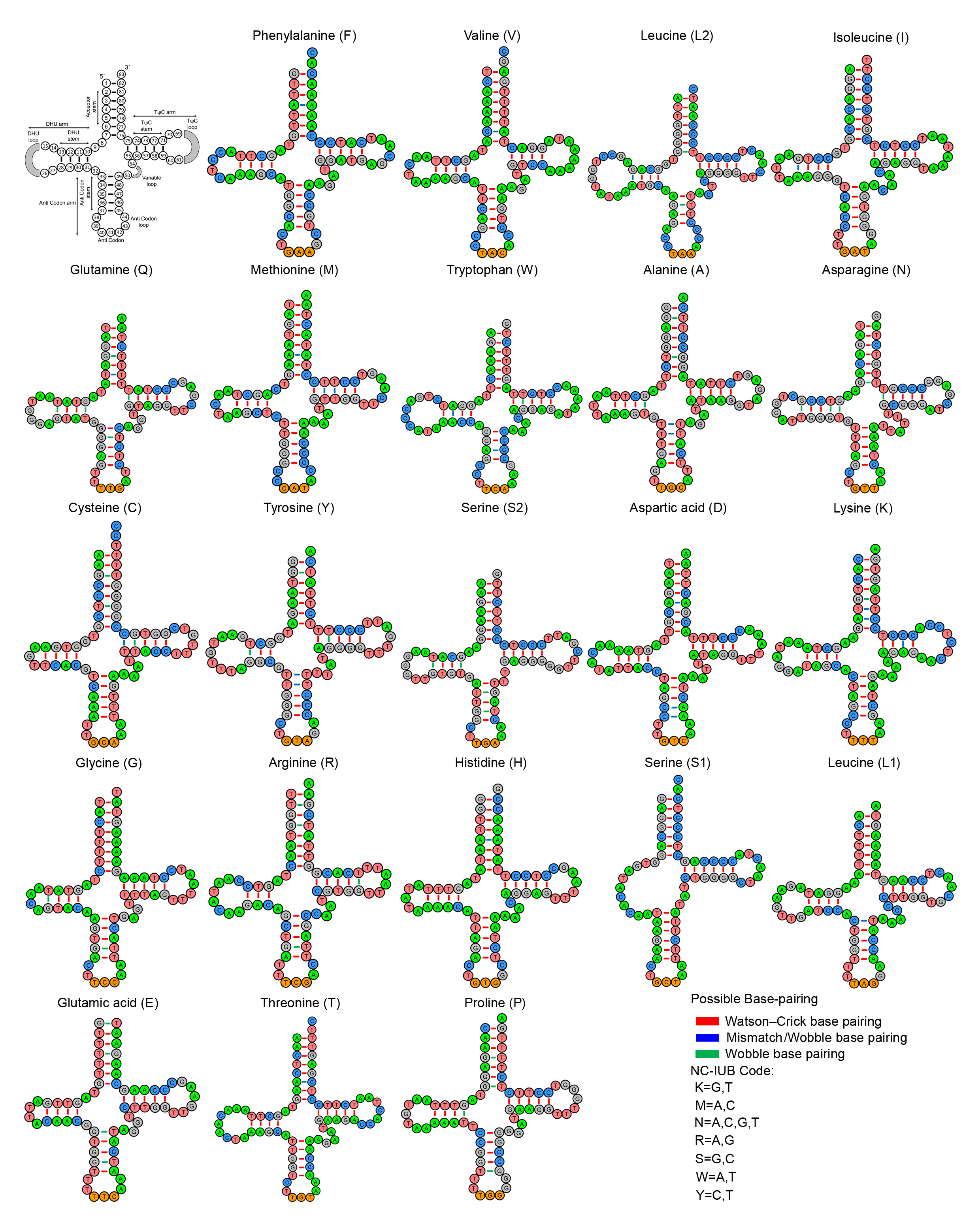

Supplement: S2 Fig — The first structure shows the nucleotide positions and details of stem-loop of tRNAs. The tRNAs are represented by full names and IUPAC-IUB single letter amino acid codes. Different base pairings are marked by red, blue and green color bars respectively. (TIF) [file pone.0225233.s002.tif]

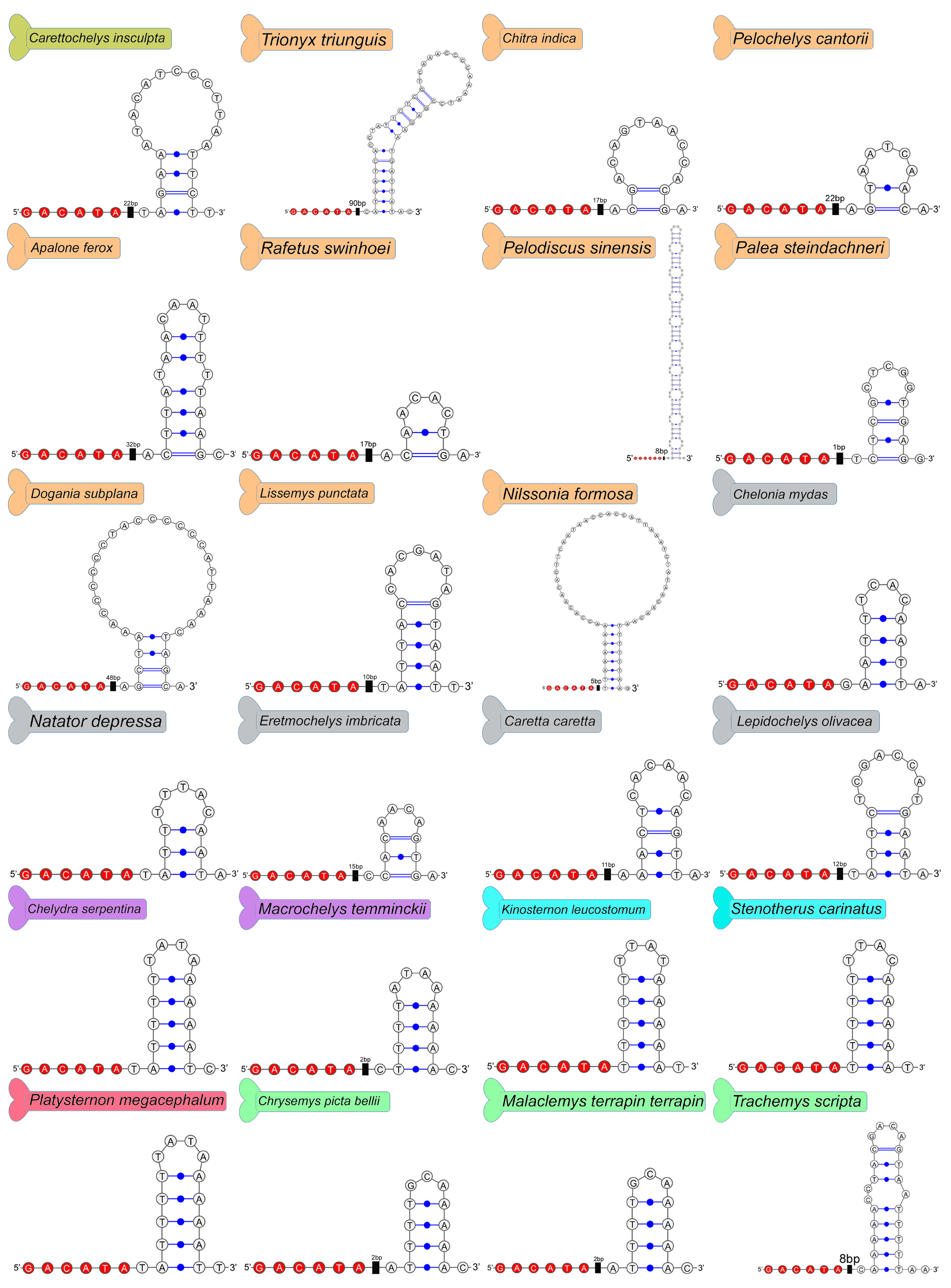

Supplement: S3 Fig — (TIF) [file pone.0225233.s003.tif]

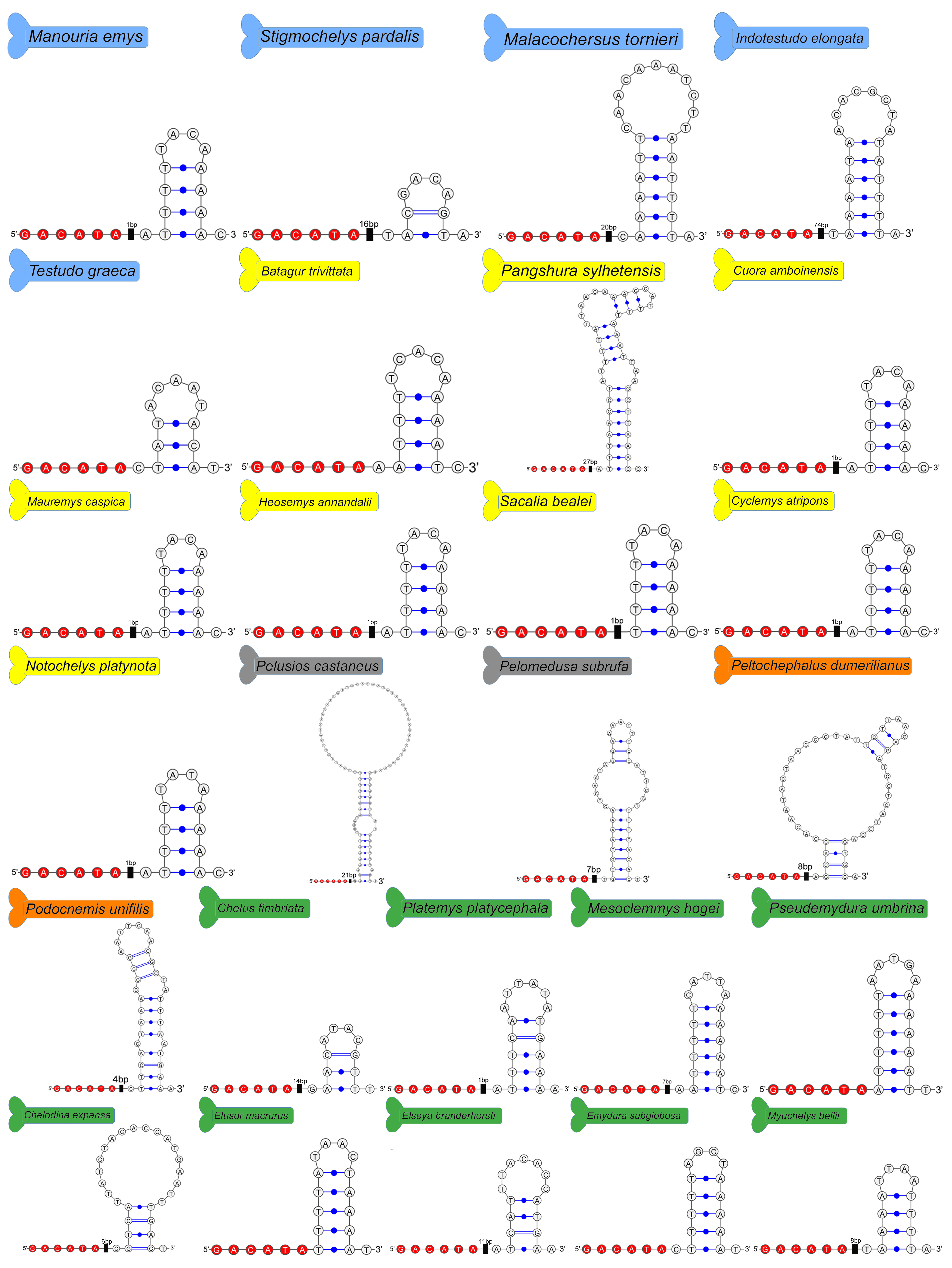

Supplement: S4 Fig — (TIF) [file pone.0225233.s004.tif]

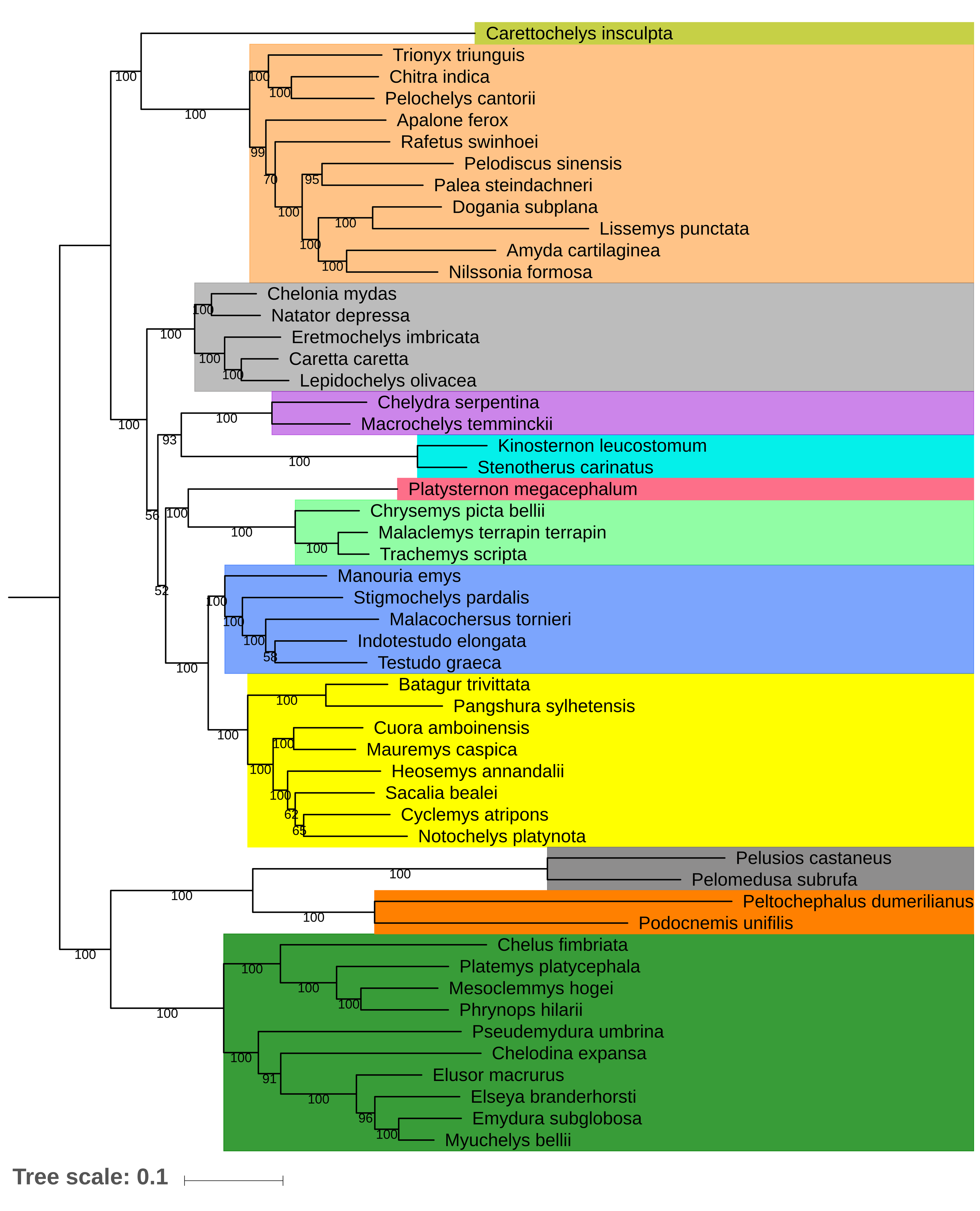

Supplement: S5 Fig — Color boxes indicate the family level clustering for the studied species. The ML tree is drawn by IQ-Tree with bootstrap support values were indicated along with each node. (TIF) [file pone.0225233.s005.tif]

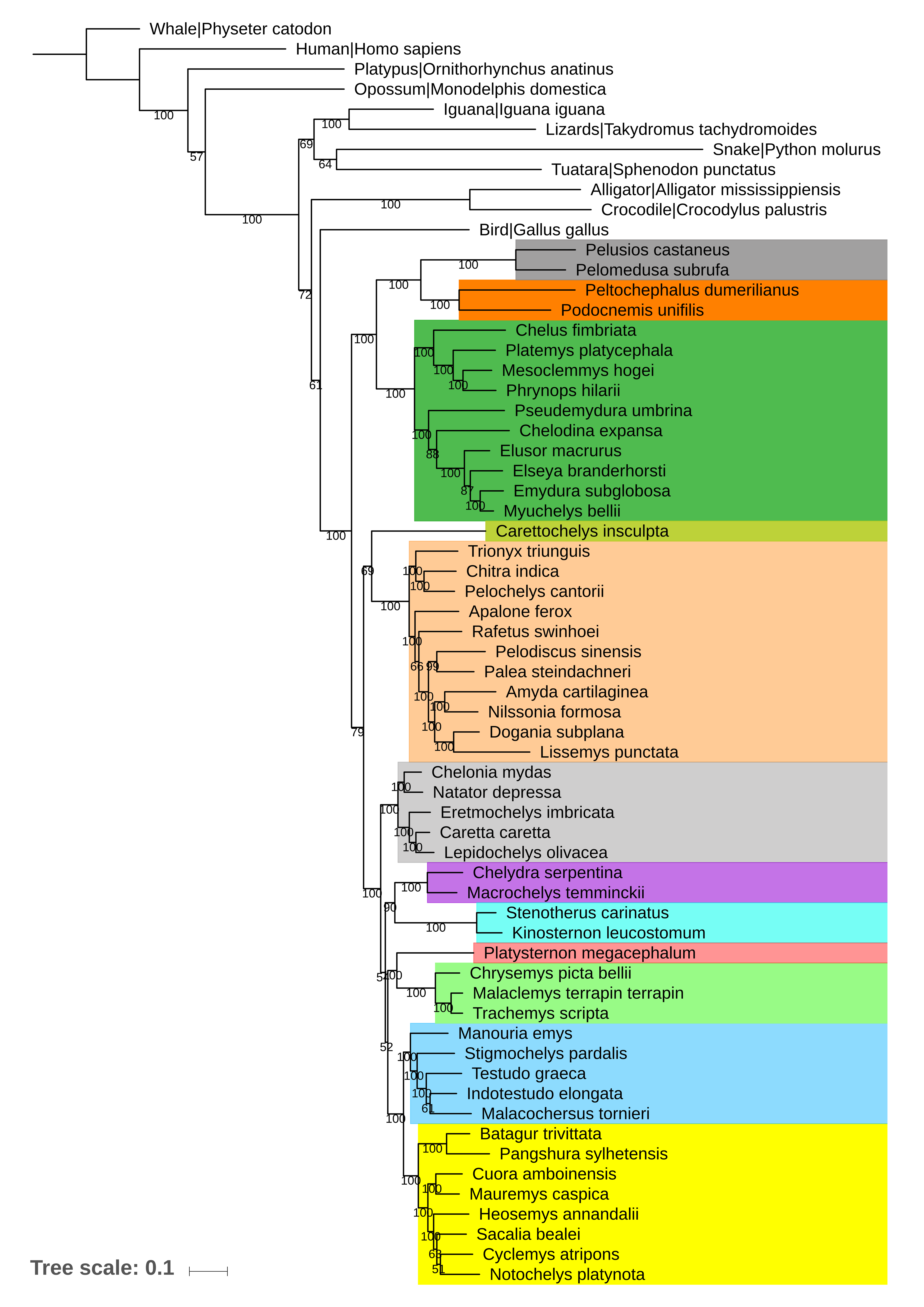

Supplement: S6 Fig — Color boxes indicate the family level clustering for the studied Testudines species. The ML tree is drawn by IQ-Tree with bootstrap support values were indicated along with each node. (TIF) [file pone.0225233.s006.tif]
