# Supplementary material for: The complete mitochondrial genome of the endangered Assam Roofed Turtle, Pangshura sylhetensis (Testudines: Geoemydidae): Genomic features and phylogeny
Source: PLoS One. 2020 Apr 23;15(4):e0225233. doi: 10.1371/journal.pone.0225233 (PMC7179895; doi:10.1371/journal.pone.0225233)
Supplement: S1 Table — (DOC) [file pone.0225233.s007.doc]

**Table S1** List of mitogenome sequences of Testudines and other amniotes species acquired from the NCBI database.

| **Sl. No.** | **Suborder** | **Family** | **Genus** | **Species** | **Accession No.** |
| --- | --- | --- | --- | --- | --- |
| 1 | Cryptodira | Geoemydidae | *Pangshura* | *P. sylhetensis* | This Study |
| 2 | Cryptodira | Geoemydidae | *Batagur* | *B. trivittata* | KX817298 |
| 3 | Cryptodira | Geoemydidae | *Cuora* | *C. amboinensis* | FJ763736 |
| 4 | Cryptodira | Geoemydidae | *Cyclemys* | *C. atripons* | EF067858 |
| 5 | Cryptodira | Geoemydidae | *Heosemys* | *H. annandalii* | JF742646 |
| 6 | Cryptodira | Geoemydidae | *Mauremys* | *M. caspica* | KC692465 |
| 7 | Cryptodira | Geoemydidae | *Notochelys* | *N. platynota* | HQ853256 |
| 8 | Cryptodira | Geoemydidae | *Sacalia* | *S. bealei* | GU183364 |
| 9 | Cryptodira | Trionychidae | *Amyda* | *A. cartilaginea* | KY100866 |
| 10 | Cryptodira | Trionychidae | *Apalone* | *A. ferox* | FJ890514 |
| 11 | Cryptodira | Trionychidae | *Chitra* | *C. indica* | JQ406951 |
| 12 | Cryptodira | Trionychidae | *Dogania* | *D. subplana* | AF366350 |
| 13 | Cryptodira | Trionychidae | *Lissemys* | *L. punctata* | EF050073 |
| 14 | Cryptodira | Trionychidae | *Nilssonia* | *N. formosa* | KT023012 |
| 15 | Cryptodira | Trionychidae | *Palea* | *P. steindachneri* | FJ541030 |
| 16 | Cryptodira | Trionychidae | *Pelochelys* | *P. cantorii* | JF719809 |
| 17 | Cryptodira | Trionychidae | *Pelodiscus* | *P. sinensis* | AY687385 |
| 18 | Cryptodira | Trionychidae | *Rafetus* | *R. swinhoei* | HQ709384 |
| 19 | Cryptodira | Trionychidae | *Trionyx* | *T. triunguis* | AB477345 |
| 20 | Cryptodira | Testudinidae | *Indotestudo* | *I. elongata* | DQ080043 |
| 21 | Cryptodira | Testudinidae | *Malacochersus* | *M. tornieri* | DQ080042 |
| 22 | Cryptodira | Testudinidae | *Manouria* | *M. emys* | DQ080040 |
| 23 | Cryptodira | Testudinidae | *Stigmochelys* | *S. pardalis* | DQ080041 |
| 24 | Cryptodira | Testudinidae | *Testudo* | *T. graeca* | DQ080049 |
| 25 | Cryptodira | Cheloniidae | *Caretta* | *C. caretta* | FR694649 |
| 26 | Cryptodira | Cheloniidae | *Chelonia* | *C. mydas* | AB012104 |
| 27 | Cryptodira | Cheloniidae | *Eretmochelys* | *E. imbricata* | DQ533485 |
| 28 | Cryptodira | Cheloniidae | *Lepidochelys* | *L. olivacea* | JX454991 |
| 29 | Cryptodira | Cheloniidae | *Natator* | *N. depressa* | JX454975 |
| 30 | Cryptodira | Chelydridae | *Chelydra* | *C. serpentina* | EF122793 |
| 31 | Cryptodira | Chelydridae | *Macrochelys* | *M. temminckii* | EF071948 |
| 32 | Cryptodira | Emydidae | *Chrysemys* | *C. picta bellii* | KF874616 |
| 33 | Cryptodira | Emydidae | *Malaclemys* | *M. terrapin terrapin* | KX774423 |
| 34 | Cryptodira | Emydidae | *Trachemys* | *T. scripta* | FJ392294 |
| 35 | Cryptodira | Kinosternidae | *Kinosternon* | *K. leucostomum* | FJ915117 |
| 36 | Cryptodira | Kinosternidae | *Stenotherus* | *S. carinatus* | HQ114563 |
| 37 | Cryptodira | Carettochelyidae | *Carettochelys* | *C. insculpta* | FJ862792 |
| 38 | Cryptodira | Platysternidae | *Platysternon* | *P. megacephalum* | DQ256377 |
| 39 | Pleurodira | Chelidae | *Chelodina* | *C. expansa* | KY705230 |
| 40 | Pleurodira | Chelidae | *Chelus* | *C. fimbriata* | HQ172156 |
| 41 | Pleurodira | Chelidae | *Elseya* | *E. branderhorsti* | KC692461 |
| 42 | Pleurodira | Chelidae | *Elusor* | *E. macrurus* | KU736930 |
| 43 | Pleurodira | Chelidae | *Emydura* | *E. subglobosa* | KC692462 |
| 44 | Pleurodira | Chelidae | *Mesoclemmys* | *M. hogei* | MF615513 |
| 45 | Pleurodira | Chelidae | *Myuchelys* | *M. bellii* | KY924930 |
| 46 | Pleurodira | Chelidae | *Platemys* | *P. platycephala* | KC692464 |
| 47 | Pleurodira | Chelidae | *Pseudemydura* | *P. umbrina* | KY486272 |
| 48 | Pleurodira | Chelidae | *Phrynops* | *P. hilarii* | JN999705 |
| 49 | Pleurodira | Pelomedusidae | *Pelomedusa* | *P. subrufa* | AF039066 |
| 50 | Pleurodira | Pelomedusidae | *Pelusios* | *P. castaneus* | KC692463 |
| 51 | Pleurodira | Podocnemididae | *Peltochephalus* | *P. dumerilianus* | AB970731 |
| 52 | Pleurodira | Podocnemididae | *Podocnemis* | *P. unifilis* | JF802204 |
| 53 | - | [Hominidae](https://www.ncbi.nlm.nih.gov/Taxonomy/Browser/wwwtax.cgi?mode=Undef&id=9604&lvl=3&lin=f&keep=1&srchmode=1&unlock) | *Homo* | *H. sapiens* | AP008580 |
| 54 | - | [Alligatoridae](https://www.ncbi.nlm.nih.gov/Taxonomy/Browser/wwwtax.cgi?mode=Undef&id=1294636&lvl=3&keep=1&srchmode=1&unlock) | *Alligator* | *A. mississippiensis* | NC_001922 |
| 55 | - | [Phasianidae](https://www.ncbi.nlm.nih.gov/Taxonomy/Browser/wwwtax.cgi?mode=Undef&id=9005&lvl=3&lin=f&keep=1&srchmode=1&unlock) | *Gallus* | *G. gallus* | AP003580 |
| 56 | - | [Pythonidae](https://www.ncbi.nlm.nih.gov/Taxonomy/Browser/wwwtax.cgi?mode=Undef&id=34984&lvl=3&keep=1&srchmode=1&unlock) | *Python* | *P. molurus* | HM581978 |
| 57 | - | [Crocodylidae](https://www.ncbi.nlm.nih.gov/Taxonomy/Browser/wwwtax.cgi?mode=Undef&id=8493&lvl=3&keep=1&srchmode=1&unlock) | *Crocodylus* | *C. palustris* | HM488007 |
| 58 | - | Iguanidae | *Iguana* | *I. iguana* | NC_002793 |
| 59 | - | Sphenodontidae | *Sphenodon* | *S. punctatus* | AF534390 |
| 60 | - | Didelphidae | *Monodelphis* | *M. domestica* | AJ508398 |
| 61 | - | Ornithorhynchidae | *Ornithorhynchus* | *O. anatinus* | NC_000891 |
| 62 | - | Lacertidae | *Takydromus* | *T. tachydromoides* | AB080237 |
| 63 | - | Physeteridae | *Physeter* | *P. catodon* | KU891394 |
