# Supplementary material for: The complete mitochondrial genome of the endangered Assam Roofed Turtle, Pangshura sylhetensis (Testudines: Geoemydidae): Genomic features and phylogeny
Source: PLoS One. 2020 Apr 23;15(4):e0225233. doi: 10.1371/journal.pone.0225233 (PMC7179895; doi:10.1371/journal.pone.0225233)
Supplement: S2 Table — (DOC) [file pone.0225233.s008.doc]

**Table S2** Estimated models by partitioning the 13 PCGs separately through PartitionFinder 2 for phylogenetic analysis of two datasets (52 mitogenomes of Testudines and 63 mitogenomes of Testudines + other amniotes).

| **Sl. No.** | **Model** | **PCGs** |
| --- | --- | --- |
| **52 mitogenomes dataset** | | |
| 1 | GTR+I+G | nad3codon1, nad1codon1, atp6codon1, cytbcodon1 |
| 2 | GTR+I+G | cox2codon2, atp6codon2, cox3codon2, cytbcodon2, nad1codon2 |
| 3 | TRN+G | nad4Lcodon3, nad3codon3, nad4codon3, atp6codon3 |
| 4 | GTR+I+G | nad4Lcodon1, nad2codon1, nad5codon1, atp8codon1, nad4codon1 |
| 5 | GTR+G | nad4Lcodon2, atp8codon2, nad3codon2, nad4codon2, nad2codon2, nad5codon2 |
| 6 | TVM+G | cox3codon3, atp8codon3, nad1codon3, nad2codon3, nad5codon3 |
| 7 | SYM+I+G | cox1codon1, cox3codon1, cox2codon1 |
| 8 | HKY+I+G | cox1codon2 |
| 9 | TVM+G | cox2codon3, cox1codon3 |
| 10 | TVM+G | cytbcodon3 |
| 11 | TVM+G | nad6codon1 |
| 12 | K81UF+G | nad6codon2 |
| 13 | HKY+G | nad6codon3 |
| **63 mitogenomes dataset** | | |
| 1 | GTR+I+G | nad4codon1, atp6codon1, nad3codon1, nad4Lcodon1 |
| 2 | GTR+I+G | cox2codon2, atp6codon2, nad1codon2, cytbcodon2, cox3codon2 |
| 3 | GTR+G | atp6codon3, nad3codon3, nad4Lcodon3, nad2codon3, nad4codon3, nad5codon3 |
| 4 | GTR+I+G | nad5codon1, nad2codon1, atp8codon2, atp8codon1 |
| 5 | TRN+G | atp8codon3 |
| 6 | SYM+I+G | cox1codon1 |
| 7 | TVM+I+G | cox1codon2 |
| 8 | TVM+G | cox2codon3, cox1codon3 |
| 9 | GTR+I+G | cytbcodon1, nad1codon1, cox3codon1, cox2codon1 |
| 10 | TVM+G | nad1codon3, cox3codon3 |
| 11 | K81UF+G | cytbcodon3 |
| 12 | GTR+I+G | nad2codon2, nad5codon2, nad3codon2, nad4codon2 |
| 13 | GTR+G | nad4Lcodon2, nad6codon2 |
| 14 | TVM+I+G | nad6codon1 |
| 15 | TVM+I+G | nad6codon3 |
