# Supplementary material for: The complete mitochondrial genome of the endangered Assam Roofed Turtle, Pangshura sylhetensis (Testudines: Geoemydidae): Genomic features and phylogeny
Source: PLoS One. 2020 Apr 23;15(4):e0225233. doi: 10.1371/journal.pone.0225233 (PMC7179895; doi:10.1371/journal.pone.0225233)
Supplement: S3 Table — (DOC) [file pone.0225233.s009.doc]

**Table S3 Gene arrangements of the studied Testudines species used in the TreeREx analysis.**

| >*P. sylhetensis*  cox1 -S1 D cox2 K atp8 atp6 cox3 G nad3 R nad4l nad4 H S2 L2 nad5 -nad6 -E cytb T -P CR F 12S V 16S nad1 I -Q M nad2 W -A -N -C -Y  >*B. trivitta*  cox1 -S1 D cox2 K atp8 atp6 cox3 G nad3 R nad4l nad4 H S2 L2 nad5 -nad6 -E cytb T -P CR F 12S V 16S nad1 I -Q M nad2 W -A -N -C -Y  >*C. amboinensis*  cox1 -S1 D cox2 K atp8 atp6 cox3 G nad3 R nad4l nad4 H S2 L2 nad5 -nad6 -E cytb T -P CR F 12S V 16S nad1 I -Q M nad2 W -A -N -C -Y  >*M. caspica*  cox1 -S1 D cox2 K atp8 atp6 cox3 G nad3 R nad4l nad4 H S2 L2 nad5 -nad6 -E cytb T -P CR F 12S V 16S nad1 I -Q M nad2 W -A -N -C -Y  >*C. atripons*  cox1 -S1 D cox2 K atp8 atp6 cox3 G nad3 R nad4l nad4 H S2 L2 nad5 -nad6 -E cytb T -P CR F 12S V 16S nad1 I -Q M nad2 W -A -N -C -Y  >*N. platynota*  cox1 -S1 D cox2 K atp8 atp6 cox3 G nad3 R nad4l nad4 H S2 L2 nad5 -nad6 -E cytb T -P CR F 12S V 16S nad1 I -Q M nad2 W -A -N -C -Y  >*H. annadali*  cox1 -S1 D cox2 K atp8 atp6 cox3 G nad3 R nad4l nad4 H S2 L2 nad5 -nad6 -E cytb T -P CR F 12S V 16S nad1 I -Q M nad2 W -A -N -C -Y  >*S. bealei*  cox1 -S1 D cox2 K atp8 atp6 cox3 G nad3 R nad4l nad4 H S2 L2 nad5 -nad6 -E cytb T -P CR F 12S V 16S nad1 I -Q M nad2 W -A -N -C -Y  >*I. elogata*  cox1 S1 D cox2 K atp8 atp6 cox3 G nad3 R nad4l nad4 H S2 L2 nad5 -nad6 -E cytb T P CR F 12S V 16S nad1 I -Q M nad2 W -A -N -C -Y  >*M. tornieri*  cox1 S1 D cox2 K atp8 atp6 cox3 G nad3 R nad4l nad4 H S2 L2 nad5 -nad6 -E cytb T P CR F 12S V 16S nad1 I -Q M nad2 W -A -N -C -Y  >*T. graeca*  cox1 S1 D cox2 K atp8 atp6 cox3 G nad3 R nad4l nad4 H S2 L2 nad5 -nad6 -E cytb T P CR F 12S V 16S nad1 I -Q M nad2 W -A -N -C -Y  >*S. pardalis*  cox1 S1 D cox2 K atp8 atp6 cox3 G nad3 R nad4l nad4 H S2 L2 nad5 -nad6 -E cytb T P CR F 12S V 16S nad1 I -Q M nad2 W -A -N -C -Y  >*M. emys*  cox1 S1 D cox2 K atp8 atp6 cox3 G nad3 R nad4l nad4 H S2 L2 nad5 -nad6 -E cytb T P CR F 12S V 16S nad1 I -Q M nad2 W -A -N -C -Y  >*C. picta belli*  cox1 -S1 D cox2 K atp8 atp6 cox3 G nad3 R nad4l nad4 H S2 L2 nad5 -nad6 -E cytb T -P CR F 12S V 16S nad1 I -Q M nad2 W -A -N -C -Y  >*M. terrapin terrapin*  cox1 -S1 D cox2 K atp8 atp6 cox3 G nad3 R nad4l nad4 H S2 L2 nad5 -nad6 -E cytb T -P CR F 12S V 16S nad1 I -Q M nad2 W -A -N -C -Y  >*T. scripta*  cox1 -S1 D cox2 K atp8 atp6 cox3 G nad3 R nad4l nad4 H S2 L2 nad5 -nad6 -E cytb T -P CR F 12S V 16S nad1 I -Q M nad2 W -A -N -C -Y  >*P. megacephalum*  cox1 S1 D cox2 K atp8 atp6 cox3 G nad3 R nad4l nad4 -nad6 -E cytb CR F 12S V 16S nad1 I H S2 L2 nad5 T P -Q M nad2 W -A -N -C -Y  >*C. serpentina*  cox1 -S1 D cox2 K atp8 atp6 cox3 G nad3 R nad4l nad4 H S2 L2 nad5 -nad6 -E cytb T -P CR F 12S V 16S nad1 I -Q M nad2 W -A -N -C -Y  >*M. temminckii*  cox1 -S1 D cox2 K atp8 atp6 cox3 G nad3 R nad4l nad4 H S2 L2 nad5 -nad6 -E cytb T -P CR F 12S V 16S nad1 I -Q M nad2 W -A -N -C -Y  >*K. leucostomum*  cox1 -S1 D cox2 K atp8 atp6 cox3 G nad3 R nad4l nad4 H S2 L2 nad5 -nad6 -E cytb T -P CR F 12S V 16S nad1 I -Q M nad2 W -A -N -C -Y  >*S. carinatus*  cox1 -S1 D cox2 K atp8 atp6 cox3 G nad3 R nad4l nad4 H S2 L2 nad5 -nad6 -E cytb T -P CR F 12S V 16S nad1 I -Q M nad2 W -A -N -C -Y  >*C. caretta*  cox1 -S1 D cox2 K atp8 atp6 cox3 G nad3 R nad4l nad4 H S2 L2 nad5 -nad6 -E cytb T -P CR F 12S V 16S nad1 I -Q M nad2 W -A -N -C -Y  >*L. olivacea*  cox1 -S1 D cox2 K atp8 atp6 cox3 G nad3 R nad4l nad4 H S2 L2 nad5 -nad6 -E cytb T -P CR F 12S V 16S nad1 I -Q M nad2 W -A -N -C -Y  >*E. imbricata*  cox1 -S1 D cox2 K atp8 atp6 cox3 G nad3 R nad4l nad4 H S2 L2 nad5 -nad6 -E cytb T P CR F 12S V 16S nad1 I -Q M nad2 W -A -N -C -Y  >*C. mydas*  cox1 -S1 D cox2 K atp8 atp6 cox3 G nad3 R nad4l nad4 H S2 L2 nad5 -nad6 -E cytb T -P CR F 12S V 16S nad1 I -Q M nad2 W -A -N -C -Y  >*N. depressa*  cox1 -S1 D cox2 K atp8 atp6 cox3 G nad3 R nad4l nad4 H S2 L2 nad5 -nad6 -E cytb T -P CR F 12S V 16S nad1 I -Q M nad2 W -A -N -C -Y  >*C. insculpta*  cox1 -S1 D cox2 K atp8 atp6 cox3 G nad3 R nad4l nad4 H S2 L2 nad5 -nad6 -E cytb T -P CR F 12S V 16S nad1 I -Q M nad2 W -A -N -C -Y  >*C. indica*  cox1 -S1 D cox2 K atp8 atp6 cox3 G nad3 R nad4l nad4 H S2 L2 nad5 -nad6 -E cytb T -P CR F 12S V 16S nad1 I -Q M nad2 W -A -N -C -Y  >*P. cantorii*  cox1 -S1 D cox2 K atp8 atp6 cox3 G nad3 R nad4l nad4 H S2 L2 nad5 -nad6 -E cytb T -P CR F 12S V 16S nad1 I -Q M nad2 W -A -N -C -Y  >*T. triunguis*  cox1 -S1 D cox2 K atp8 atp6 cox3 G nad3 R nad4l nad4 H S2 L2 nad5 -nad6 -E cytb T -P CR F 12S V 16S nad1 I -Q M nad2 W -A -N -C -Y  >*A. ferox*  cox1 -S1 D cox2 K atp8 atp6 cox3 G nad3 R nad4l nad4 H S2 L2 nad5 -nad6 -E cytb T -P CR F 12S V 16S nad1 I -Q M nad2 W -A -N -C -Y  >*R. swinhoei*  cox1 -S1 D cox2 K atp8 atp6 cox3 G nad3 R nad4l nad4 H S2 L2 nad5 -nad6 -E cytb T -P CR F 12S V 16S nad1 I -Q M nad2 W -A -N -C -Y  >*P. sinensis*  cox1 -S1 D cox2 K atp8 atp6 cox3 G nad3 R nad4l nad4 H S2 L2 nad5 -nad6 -E cytb T -P CR F 12S V 16S nad1 I -Q M nad2 W -A -N -C -Y  >*P. steindachneri*  cox1 -S1 D cox2 K atp8 atp6 cox3 G nad3 R nad4l nad4 H S2 L2 nad5 -nad6 -E cytb T -P CR F 12S V 16S nad1 I -Q M nad2 W -A -N -C -Y  >*D. subplana*  cox1 -S1 D cox2 K atp8 atp6 cox3 G nad3 R nad4l nad4 H S2 L2 nad5 -nad6 -E cytb T -P CR F 12S V 16S nad1 I -Q M nad2 W -A -N -C -Y  >*L. punctata*  cox1 -S1 D cox2 K atp8 atp6 cox3 G nad3 R nad4l nad4 H S2 L2 nad5 -nad6 -E cytb T -P CR F 12S V 16S nad1 I -Q M nad2 W -A -N -C -Y  >*A. cartilaginea*  cox1 -S1 D cox2 K atp8 atp6 cox3 G nad3 R nad4l nad4 H S2 L2 nad5 -nad6 -E cytb T -P CR F 12S V 16S nad1 I -Q M nad2 W -A -N -C -Y  >*N. formosa*  cox1 -S1 D cox2 K atp8 atp6 cox3 G nad3 R nad4l nad4 H S2 L2 nad5 -nad6 -E cytb T -P CR F 12S V 16S nad1 I -Q M nad2 W -A -N -C -Y  >*C. expansa*  cox1 -S1 D cox2 K atp8 atp6 cox3 G nad3 R nad4l nad4 H S2 L2 nad5 -nad6 -E cytb T -P CR F 12S V 16S nad1 I -Q M nad2 W -A -N -C -Y  >*E. branderhorsti*  cox1 -S1 D cox2 K atp8 atp6 cox3 G nad3 R nad4l nad4 H S2 L2 nad5 -nad6 -E cytb T -P CR F 12S V 16S nad1 I -Q M nad2 W -A -N -C -Y  >*E. subglobosa*  cox1 -S1 D cox2 K atp8 atp6 cox3 G nad3 R nad4l nad4 H S2 L2 nad5 -nad6 -E cytb T -P CR F 12S V 16S nad1 I -Q M nad2 W -A -N -C -Y  >*M. bellii*  cox1 -S1 D cox2 K atp8 atp6 cox3 G nad3 R nad4l nad4 H S2 L2 nad5 -nad6 -E cytb T -P CR F 12S V 16S nad1 I -Q M nad2 W -A -N -C -Y  >*E. macrurus*  cox1 -S1 D cox2 K atp8 atp6 cox3 G nad3 R nad4l nad4 H S2 L2 nad5 -nad6 -E cytb T P CR F 12S V 16S nad1 I -Q M nad2 W -A -N -C -Y  >*P. umbrina*  cox1 -S1 D cox2 K atp8 atp6 cox3 G nad3 R nad4l nad4 H S2 L2 nad5 -nad6 -E cytb T -P CR F 12S V 16S nad1 I -Q M nad2 W -A -N -C -Y  >*C. fimbriata*  cox1 -S1 D cox2 K atp8 atp6 cox3 G nad3 R nad4l nad4 H S2 L2 nad5 -nad6 -E cytb T -P CR F 12S V 16S nad1 I -Q M nad2 W -A -N -C -Y  >*M. hogei*  cox1 -S1 D cox2 K atp8 atp6 cox3 G nad3 R nad4l nad4 H S2 L2 nad5 -nad6 -E cytb T -P CR F 12S V 16S nad1 I -Q M nad2 W -A -N -C -Y  >*P. hilarii*  cox1 -S1 D cox2 K atp8 atp6 cox3 G nad3 R nad4l nad4 H S2 L2 nad5 -nad6 -E cytb T -P CR F 12S V 16S nad1 I -Q M nad2 W -A -N -C -Y  >*P. platycephala*  cox1 -S1 D cox2 K atp8 atp6 cox3 G nad3 R nad4l nad4 H S2 L2 nad5 -nad6 -E cytb T -P CR F 12S V 16S nad1 I -Q M nad2 W -A -N -C -Y  >*P. castaneus*  cox1 -S1 D cox2 K atp8 atp6 cox3 G nad3 R nad4l nad4 H S2 L2 nad5 -nad6 -E cytb T -P CR F 12S V 16S nad1 I -Q M nad2 W -A -N -C -Y  >*P. subrufa*  cox1 -S1 D cox2 K atp8 atp6 cox3 G nad3 R nad4l nad4 H S2 L2 nad5 -nad6 -E cytb T -P CR F 12S V 16S nad1 I -Q M nad2 W -A -N -C -Y  >*P. dumerilianus*  cox1 -S1 D cox2 K atp8 atp6 cox3 G nad3 R nad4l nad4 H S2 L2 nad5 -nad6 -E cytb T -P CR F 12S V 16S nad1 I -Q M nad2 W -A -N -C -Y  >*P. unifilis*  cox1 -S1 D cox2 K atp8 atp6 cox3 G nad3 R nad4l nad4 H S2 L2 nad5 -nad6 -E cytb T -P CR F 12S V 16S nad1 I -Q M nad2 W -A -N -C -Y |
| --- |
