# Supplementary material for: The complete mitochondrial genome of the endangered Assam Roofed Turtle, Pangshura sylhetensis (Testudines: Geoemydidae): Genomic features and phylogeny
Source: PLoS One. 2020 Apr 23;15(4):e0225233. doi: 10.1371/journal.pone.0225233 (PMC7179895; doi:10.1371/journal.pone.0225233)
Supplement: S4 Table — The A+T biases of whole mitogenome, PCGs, tRNAs, rRNAs, and CRs were calculated by AT-skew = (A-T)/(A+T) and GC-skew = (G-C)/(G+C), respectively. (DOC) [file pone.0225233.s010.doc]

**Table S4** Nucleotide composition of 52 Testudines species mitochondrial genomes. The A+T biases of whole mitogenome, protein coding genes, tRNA, rRNA, and control regions were calculated by AT-skew = (A-T)/(A+T) and GC-skew= (G-C)/(G+C), respectively.

| **Species** | **Size(bp)** | **A%** | **T%** | **G%** | **C%** | **A+T%** | **G+C%** | **AT-Skew** | **GC- Skew** |
| --- | --- | --- | --- | --- | --- | --- | --- | --- | --- |
| **Complete mitogenome** | | | | | | | | | |
| *P. sylhetensis* | 16568 | 33.32 | 25.94 | 13.55 | 27.17 | 59.27 | 40.72 | 0.124 | -0.334 |
| *B. trivittata* | 16463 | 33.60 | 24.52 | 13.25 | 28.62 | 58.12 | 41.87 | 0.156 | -0.366 |
| *C. amboinensis* | 16708 | 33.82 | 26.74 | 13.05 | 26.36 | 60.57 | 39.42 | 0.116 | -0.337 |
| *C. atripons* | 16500 | 34.42 | 27.20 | 13.01 | 25.36 | 61.62 | 38.37 | 0.117 | -0.321 |
| *H. annandalii* | 16604 | 35.14 | 26.71 | 12.27 | 25.87 | 61.85 | 38.14 | 0.136 | -0.356 |
| *M. caspica* | 16741 | 34.04 | 27.17 | 12.91 | 25.87 | 61.21 | 38.78 | 0.112 | -0.334 |
| *N. platynota* | 16981 | 34.39 | 28.10 | 12.24 | 25.25 | 62.49 | 37.50 | 0.100 | -0.347 |
| *S. bealei* | 16561 | 34.18 | 26.86 | 13.06 | 25.88 | 61.04 | 38.95 | 0.119 | -0.329 |
| *A. cartilaginea* | 15339 | 36.36 | 25.13 | 11.97 | 26.52 | 61.49 | 38.50 | 0.182 | -0.377 |
| *A. ferox* | 16866 | 35.80 | 25.38 | 11.82 | 26.98 | 61.18 | 38.81 | 0.170 | -0.390 |
| *C. indica* | 16726 | 35.79 | 24.33 | 11.87 | 27.99 | 60.12 | 39.87 | 0.190 | -0.404 |
| *D. subplana* | 17289 | 35.29 | 25.96 | 11.94 | 26.78 | 61.26 | 38.73 | 0.152 | -0.383 |
| *L. punctata* | 16489 | 35.73 | 25.68 | 12.22 | 26.36 | 61.41 | 38.58 | 0.163 | -0.366 |
| *N. formosa* | 17145 | 37.13 | 25.83 | 11.19 | 25.82 | 62.97 | 37.02 | 0.179 | -0.395 |
| *P. steindachneri* | 17243 | 34.85 | 26.51 | 12.28 | 26.34 | 61.36 | 38.63 | 0.135 | -0.363 |
| *P. cantorii* | 17499 | 35.24 | 24.21 | 11.98 | 28.54 | 59.46 | 40.53 | 0.185 | -0.408 |
| *P. sinensis* | 17364 | 35.22 | 27.26 | 11.77 | 25.73 | 62.49 | 37.50 | 0.127 | -0.372 |
| *R. swinhoei* | 16990 | 34.98 | 25.46 | 12.24 | 27.30 | 60.45 | 39.54 | 0.157 | -0.380 |
| *T. triunguis* | 16590 | 35.33 | 23.14 | 12.20 | 29.31 | 58.48 | 41.51 | 0.208 | -0.412 |
| *I. elongata* | 16788 | 35.30 | 26.46 | 12.08 | 26.14 | 61.77 | 38.22 | 0.143 | -0.368 |
| *M. tornieri* | 19202 | 35.06 | 27.10 | 11.15 | 26.67 | 62.17 | 37.82 | 0.128 | -0.410 |
| *M. emys* | 16455 | 34.10 | 25.86 | 13.18 | 26.84 | 59.96 | 40.03 | 0.137 | -0.341 |
| *S. pardalis* | 19403 | 32.27 | 26.63 | 14.45 | 26.64 | 58.90 | 41.09 | 0.095 | -0.296 |
| *T. graeca* | 19278 | 34.09 | 28.62 | 11.78 | 25.50 | 62.71 | 37.28 | 0.087 | -0.367 |
| *C. caretta* | 16637 | 35.37 | 25.90 | 11.96 | 26.75 | 61.28 | 38.71 | 0.154 | -0.382 |
| *C. mydas* | 16497 | 35.39 | 25.15 | 11.93 | 27.51 | 60.55 | 39.44 | 0.169 | -0.394 |
| *E. imbricata* | 16478 | 35.41 | 25.57 | 12.07 | 26.93 | 60.99 | 39.00 | 0.161 | -0.381 |
| *L. olivacea* | 16718 | 35.48 | 26.58 | 11.84 | 26.07 | 62.07 | 37.92 | 0.143 | -0.375 |
| *N. depressa* | 16281 | 35.32 | 25.03 | 12.04 | 27.59 | 60.36 | 39.63 | 0.170 | -0.392 |
| *C. serpentina* | 16631 | 34.42 | 27.86 | 12.60 | 25.09 | 62.29 | 37.70 | 0.105 | -0.331 |
| *M. temminckii* | 16569 | 34.68 | 27.40 | 12.66 | 25.24 | 62.09 | 37.90 | 0.117 | -0.331 |
| *C. picta bellii* | 16875 | 34.35 | 26.62 | 12.97 | 26.04 | 60.98 | 39.01 | 0.126 | -0.335 |
| *M. terrapin terrapin* | 16717 | 33.95 | 27.03 | 13.11 | 25.89 | 60.98 | 39.01 | 0.113 | -0.327 |
| *T. scripta* | 16810 | 34.27 | 26.98 | 12.85 | 25.87 | 61.26 | 38.73 | 0.118 | -0.336 |
| *K. leucostomum* | 16559 | 35.96 | 28.22 | 12.02 | 23.77 | 64.19 | 35.80 | 0.120 | -0.328 |
| *S. carinatus* | 16554 | 35.82 | 27.30 | 11.99 | 24.87 | 63.12 | 36.87 | 0.134 | -0.349 |
| *C. insculpta* | 16439 | 36.34 | 23.79 | 11.72 | 28.13 | 60.13 | 39.86 | 0.208 | -0.411 |
| *P.megacephalum* | 19043 | 33.99 | 27.40 | 12.95 | 25.64 | 61.39 | 38.60 | 0.107 | -0.328 |
| *C. expansa* | 16500 | 35.66 | 26.43 | 12.80 | 25.10 | 62.09 | 37.90 | 0.148 | -0.324 |
| *C. fimbriata* | 16661 | 35.97 | 25.35 | 12.28 | 26.39 | 61.32 | 38.67 | 0.173 | -0.364 |
| *E. branderhorsti* | 16444 | 35.22 | 25.16 | 12.65 | 26.95 | 60.38 | 39.61 | 0.166 | -0.361 |
| *E. macrurus* | 16499 | 34.90 | 25.81 | 12.69 | 26.57 | 60.72 | 39.27 | 0.149 | -0.353 |
| *E.subglobosa* | 16442 | 34.95 | 26.63 | 12.72 | 25.68 | 61.59 | 38.40 | 0.135 | -0.337 |
| *M. hogei* | 16513 | 34.59 | 25.11 | 12.75 | 27.52 | 59.71 | 40.28 | 0.158 | -0.366 |
| *M. bellii* | 16465 | 35.33 | 25.72 | 12.39 | 26.54 | 61.06 | 38.93 | 0.157 | -0.363 |
| *P.platycephala* | 16553 | 34.57 | 25.30 | 12.55 | 27.55 | 59.88 | 40.11 | 0.154 | -0.373 |
| *P.umbrina* | 16410 | 34.82 | 28.68 | 12.51 | 23.97 | 63.51 | 36.48 | 0.096 | -0.313 |
| *P. hilarii* | 15571 | 34.00 | 23.76 | 13.58 | 28.62 | 57.76 | 42.20 | 0.177 | -0.356 |
| *P. subrufa* | 16787 | 33.91 | 27.38 | 12.09 | 26.60 | 61.29 | 38.70 | 0.106 | -0.374 |
| *P. castaneus* | 16761 | 32.37 | 27.18 | 12.21 | 28.22 | 59.56 | 40.43 | 0.087 | -0.395 |
| *P.dumerilianus* | 16601 | 35.73 | 24.10 | 12.67 | 27.48 | 59.83 | 40.16 | 0.194 | -0.368 |
| *P. unifilis* | 16493 | 33.49 | 28.00 | 12.13 | 26.35 | 61.50 | 38.49 | 0.089 | -0.369 |
| **Protein Coding genes (PCGs)** | | | | | | | | | |
| *P. sylhetensis* | 11268 | 31.06 | 27.71 | 13.48 | 27.74 | 58.77 | 41.22 | 0.056 | -0.345 |
| *B. trivittata* | 11379 | 31.37 | 26.07 | 13.05 | 29.49 | 57.44 | 42.55 | 0.092 | -0.386 |
| *C. amboinensis* | 11397 | 31.44 | 28.05 | 13.07 | 27.42 | 59.49 | 40.50 | 0.057 | -0.354 |
| *C. atripons* | 11387 | 31.88 | 29.16 | 13.05 | 25.88 | 61.05 | 38.94 | 0.044 | -0.329 |
| *H. annandalii* | 11380 | 32.65 | 28.31 | 12.12 | 26.90 | 60.96 | 39.03 | 0.071 | -0.378 |
| *M. caspica* | 11382 | 31.70 | 28.40 | 13.00 | 26.88 | 60.11 | 39.88 | 0.054 | -0.348 |
| *N. platynota* | 11398 | 32.12 | 29.47 | 12.50 | 25.89 | 61.60 | 38.39 | 0.043 | -0.348 |
| *S. bealei* | 11373 | 31.82 | 28.57 | 12.92 | 26.67 | 60.39 | 39.60 | 0.053 | -0.347 |
| *A. cartilaginea* | 11329 | 34.03 | 27.43 | 11.77 | 26.75 | 61.47 | 38.52 | 0.107 | -0.388 |
| *A. ferox* | 11350 | 33.70 | 26.52 | 11.84 | 27.92 | 60.23 | 39.76 | 0.119 | -0.404 |
| *C. indica* | 11351 | 33.66 | 25.75 | 11.84 | 28.74 | 59.41 | 40.58 | 0.133 | -0.416 |
| *D. subplana* | 11326 | 33.47 | 27.44 | 11.99 | 27.08 | 60.91 | 39.08 | 0.099 | -0.386 |
| *L. punctata* | 11333 | 33.60 | 27.57 | 11.86 | 26.95 | 61.17 | 38.82 | 0.098 | -0.388 |
| *N. formosa* | 11347 | 33.77 | 27.33 | 11.72 | 27.16 | 61.11 | 38.88 | 0.105 | -0.397 |
| *P. steindachneri* | 11212 | 33.09 | 27.82 | 11.95 | 27.12 | 60.92 | 39.07 | 0.086 | -0.388 |
| *P. cantorii* | 11348 | 32.81 | 25.04 | 12.34 | 29.79 | 57.86 | 42.13 | 0.134 | -0.414 |
| *P. sinensis* | 11376 | 33.65 | 29.30 | 11.66 | 25.36 | 62.96 | 37.03 | 0.069 | -0.370 |
| *R. swinhoei* | 11375 | 32.94 | 26.79 | 12.25 | 28.00 | 59.74 | 40.25 | 0.103 | -0.391 |
| *T. triunguis* | 11343 | 32.59 | 24.67 | 12.52 | 30.20 | 57.26 | 42.73 | 0.138 | -0.413 |
| *I. elongata* | 11379 | 33.03 | 28.10 | 11.99 | 26.86 | 61.13 | 38.86 | 0.080 | -0.382 |
| *M. tornieri* | 11379 | 33.01 | 27.72 | 11.83 | 27.41 | 60.74 | 39.25 | 0.087 | -0.396 |
| *M. emys* | 11367 | 31.69 | 27.56 | 13.28 | 27.45 | 59.25 | 40.74 | 0.069 | -0.347 |
| *S. pardalis* | 11364 | 32.58 | 27.53 | 12.58 | 27.29 | 60.11 | 39.88 | 0.084 | -0.368 |
| *T. graeca* | 11382 | 32.59 | 28.07 | 12.47 | 26.85 | 60.66 | 39.33 | 0.074 | -0.365 |
| *C. caretta* | 11370 | 33.21 | 27.14 | 11.71 | 27.92 | 60.36 | 39.63 | 0.100 | -0.408 |
| *C. mydas* | 11400 | 33.24 | 26.72 | 11.57 | 28.44 | 59.97 | 40.02 | 0.108 | -0.421 |
| *E. imbricata* | 11394 | 33.29 | 27.26 | 11.75 | 27.68 | 60.56 | 39.43 | 0.099 | -0.403 |
| *L. olivacea* | 11373 | 33.23 | 27.75 | 11.85 | 27.15 | 60.99 | 39.00 | 0.089 | -0.392 |
| *N. depressa* | 11379 | 33.23 | 26.68 | 11.67 | 28.39 | 59.92 | 40.07 | 0.109 | -0.417 |
| *C. serpentina* | 11380 | 32.46 | 29.53 | 12.35 | 25.64 | 62.00 | 37.99 | 0.047 | -0.349 |
| *M. temminckii* | 11411 | 32.45 | 29.19 | 12.65 | 25.69 | 61.65 | 38.34 | 0.052 | -0.340 |
| *C. picta bellii* | 11404 | 32.06 | 28.32 | 12.78 | 26.82 | 60.39 | 39.60 | 0.062 | -0.354 |
| *M. terrapin terrapin* | 11424 | 31.60 | 28.87 | 13.00 | 26.51 | 60.47 | 39.52 | 0.045 | -0.341 |
| *T. scripta* | 11418 | 32.02 | 28.85 | 12.67 | 26.44 | 60.88 | 39.11 | 0.052 | -0.351 |
| *K. leucostomum* | 11382 | 34.10 | 30.21 | 11.72 | 23.95 | 64.32 | 35.67 | 0.060 | -0.342 |
| *S. carinatus* | 11367 | 33.78 | 29.36 | 11.83 | 25.01 | 63.14 | 36.85 | 0.069 | -0.357 |
| *C. insculpta* | 11333 | 34.06 | 25.43 | 11.78 | 28.70 | 59.50 | 40.49 | 0.145 | -0.417 |
| *P.megacephalum* | 11395 | 30.98 | 28.81 | 13.83 | 26.36 | 59.80 | 40.19 | 0.036 | -0.311 |
| *C. expansa* | 11312 | 33.46 | 28.36 | 12.51 | 25.64 | 61.83 | 38.16 | 0.082 | -0.343 |
| *C. fimbriata* | 11289 | 33.59 | 27.02 | 12.17 | 27.21 | 60.61 | 39.38 | 0.108 | -0.381 |
| *E.branderhorsti* | 11310 | 32.76 | 27.06 | 12.68 | 27.48 | 59.83 | 40.16 | 0.095 | -0.368 |
| *E. macrurus* | 11309 | 32.36 | 27.58 | 12.75 | 27.28 | 59.95 | 40.04 | 0.079 | -0.362 |
| *E.subglobosa* | 11311 | 32.64 | 28.50 | 12.65 | 26.19 | 61.15 | 38.84 | 0.067 | -0.348 |
| *M. hogei* | 11285 | 32.25 | 26.68 | 12.49 | 28.56 | 58.93 | 41.06 | 0.094 | -0.391 |
| *M. bellii* | 11310 | 33.04 | 27.41 | 12.30 | 27.23 | 60.45 | 39.54 | 0.093 | -0.377 |
| *P.platycephala* | 11329 | 32.18 | 27.07 | 12.26 | 28.47 | 59.25 | 40.74 | 0.086 | -0.397 |
| *P.umbrina* | 11312 | 32.62 | 30.79 | 12.31 | 24.26 | 63.41 | 36.58 | 0.028 | -0.326 |
| *P. hilarii* | 11353 | 31.33 | 25.80 | 13.48 | 29.39 | 57.13 | 42.87 | 0.097 | -0.371 |
| *P. subrufa* | 11392 | 31.58 | 28.96 | 12.00 | 27.44 | 60.55 | 39.44 | 0.043 | -0.391 |
| *P. castaneus* | 11299 | 30.27 | 28.43 | 12.06 | 29.22 | 58.71 | 41.28 | 0.031 | -0.415 |
| *P.dumerilianus* | 11434 | 34.17 | 25.99 | 12.02 | 27.80 | 60.17 | 39.82 | 0.136 | -0.396 |
| *P. unifilis* | 11430 | 31.64 | 29.87 | 11.72 | 26.75 | 61.52 | 38.47 | 0.028 | -0.390 |
| **tRNA gene** | | | | | | | | | |
| *P. sylhetensis* | 1550 | 30.45 | 29.41 | 21.22 | 18.90 | 59.87 | 40.12 | 0.017 | 0.057 |
| *B. trivittata* | 1551 | 30.75 | 29.27 | 20.89 | 19.08 | 60.02 | 39.97 | 0.024 | 0.045 |
| *C. amboinensis* | 1608 | 32.46 | 30.10 | 19.21 | 18.22 | 62.56 | 37.43 | 0.037 | 0.026 |
| *C. atripons* | 1551 | 32.10 | 30.11 | 19.66 | 18.11 | 62.21 | 37.78 | 0.032 | 0.040 |
| *H. annandalii* | 1550 | 32.25 | 29.87 | 19.35 | 18.51 | 62.12 | 37.87 | 0.038 | 0.022 |
| *M. caspica* | 1554 | 32.17 | 29.66 | 19.62 | 18.53 | 61.84 | 38.16 | 0.040 | 0.028 |
| *N. platynota* | 1551 | 32.49 | 29.98 | 19.27 | 18.24 | 62.47 | 37.52 | 0.040 | 0.027 |
| *S. bealei* | 1549 | 32.08 | 29.89 | 20.01 | 18.01 | 61.97 | 38.02 | 0.035 | 0.052 |
| *A. cartilaginea* | 1407 | 32.76 | 30.06 | 18.40 | 18.76 | 62.82 | 37.17 | 0.042 | -0.009 |
| *A. ferox* | 1556 | 35.60 | 27.05 | 14.65 | 22.68 | 62.66 | 37.33 | 0.136 | -0.215 |
| *C. indica* | 1555 | 35.11 | 26.30 | 14.98 | 23.60 | 61.41 | 38.58 | 0.143 | -0.223 |
| *D. subplana* | 1540 | 35.45 | 26.62 | 14.87 | 23.05 | 62.07 | 37.92 | 0.142 | -0.215 |
| *L. punctata* | 1554 | 35.00 | 26.06 | 15.25 | 23.68 | 61.06 | 38.93 | 0.146 | -0.216 |
| *N. formosa* | 1554 | 36.16 | 27.02 | 14.67 | 22.13 | 63.19 | 36.80 | 0.144 | -0.202 |
| *P. steindachneri* | 1543 | 34.93 | 27.09 | 15.48 | 22.48 | 62.02 | 37.97 | 0.126 | -0.184 |
| *P. cantorii* | 1545 | 34.23 | 25.24 | 15.66 | 24.85 | 59.48 | 40.51 | 0.151 | -0.226 |
| *P. sinensis* | 1571 | 35.00 | 28.00 | 14.70 | 22.27 | 63.01 | 36.98 | 0.111 | -0.204 |
| *R. swinhoei* | 1540 | 34.80 | 26.62 | 15.19 | 23.37 | 61.42 | 38.57 | 0.133 | -0.212 |
| *T. triunguis* | 1541 | 35.10 | 25.43 | 15.25 | 24.20 | 60.54 | 39.45 | 0.159 | -0.227 |
| *I. elongata* | 1539 | 32.16 | 29.56 | 19.81 | 18.45 | 61.72 | 38.27 | 0.042 | 0.035 |
| *M. tornieri* | 1610 | 31.98 | 29.19 | 19.56 | 19.25 | 61.18 | 38.82 | 0.045 | 0.008 |
| *M. emys* | 1552 | 31.82 | 29.89 | 19.78 | 18.49 | 61.72 | 38.27 | 0.031 | 0.033 |
| *S. pardalis* | 1551 | 32.49 | 29.52 | 19.27 | 18.69 | 62.02 | 37.97 | 0.047 | 0.015 |
| *T. graeca* | 1545 | 31.52 | 29.32 | 20.25 | 18.90 | 60.84 | 39.15 | 0.036 | 0.034 |
| *C. caretta* | 1549 | 31.69 | 28.98 | 20.07 | 19.23 | 60.68 | 39.31 | 0.044 | 0.021 |
| *C. mydas* | 1552 | 31.82 | 29.57 | 19.84 | 18.75 | 61.40 | 38.59 | 0.036 | 0.028 |
| *E. imbricata* | 1550 | 31.74 | 28.83 | 20.32 | 19.09 | 60.58 | 39.41 | 0.047 | 0.031 |
| *L. olivacea* | 1547 | 31.86 | 28.95 | 19.91 | 19.26 | 60.82 | 39.17 | 0.047 | 0.016 |
| *N. depressa* | 1552 | 31.95 | 29.38 | 19.65 | 19.00 | 61.34 | 38.66 | 0.042 | 0.016 |
| *C. serpentina* | 1548 | 31.26 | 30.74 | 20.22 | 17.76 | 62.01 | 37.98 | 0.008 | 0.064 |
| *M. temminckii* | 1547 | 31.73 | 30.31 | 19.52 | 18.42 | 62.05 | 37.94 | 0.022 | 0.028 |
| *C. picta bellii* | 1493 | 32.61 | 29.87 | 19.29 | 18.21 | 62.49 | 37.50 | 0.043 | 0.028 |
| *M. terrapin terrapin* | 1553 | 32.26 | 29.36 | 19.76 | 18.60 | 61.62 | 38.37 | 0.047 | 0.030 |
| *T. scripta* | 1555 | 32.34 | 29.51 | 19.42 | 18.71 | 61.86 | 38.13 | 0.045 | 0.018 |
| *K. leucostomum* | 1485 | 33.06 | 29.83 | 18.78 | 18.31 | 62.89 | 37.10 | 0.051 | 0.012 |
| *S. carinatus* | 1550 | 32.90 | 29.61 | 19.22 | 18.25 | 62.51 | 37.48 | 0.052 | 0.025 |
| *C. insculpta* | 1494 | 31.72 | 29.25 | 19.88 | 19.14 | 60.97 | 39.02 | 0.040 | 0.018 |
| *P.megacephalum* | 1684 | 32.77 | 29.57 | 18.94 | 18.70 | 62.35 | 37.64 | 0.051 | 0.006 |
| *C. expansa* | 1546 | 32.27 | 29.94 | 19.27 | 18.49 | 62.22 | 37.77 | 0.037 | 0.020 |
| *C. fimbriata* | 1549 | 32.21 | 29.24 | 19.69 | 18.85 | 61.45 | 38.54 | 0.048 | 0.021 |
| *E.branderhorsti* | 1540 | 32.07 | 28.50 | 19.87 | 19.54 | 60.58 | 39.41 | 0.058 | 0.008 |
| *E. macrurus* | 1542 | 32.23 | 29.11 | 19.45 | 19.19 | 61.34 | 38.65 | 0.050 | 0.006 |
| *E.subglobosa* | 1538 | 31.92 | 29.71 | 19.76 | 18.59 | 61.63 | 38.36 | 0.035 | 0.030 |
| *M. hogei* | 1492 | 32.77 | 29.29 | 18.63 | 19.30 | 62.06 | 37.93 | 0.056 | -0.017 |
| *M. bellii* | 1541 | 32.70 | 29.13 | 19.33 | 18.81 | 61.84 | 38.15 | 0.057 | 0.013 |
| *P.platycephala* | 1555 | 32.92 | 28.93 | 18.84 | 19.29 | 61.86 | 38.13 | 0.064 | -0.011 |
| *P.umbrina* | 1480 | 32.56 | 30.74 | 18.58 | 18.10 | 63.31 | 36.68 | 0.028 | 0.012 |
| *P. hilarii* | 1524 | 32.09 | 28.74 | 19.95 | 19.23 | 60.83 | 39.17 | 0.055 | 0.018 |
| *P. subrufa* | 1530 | 32.15 | 29.73 | 19.73 | 18.36 | 61.89 | 38.10 | 0.039 | 0.036 |
| *P. castaneus* | 1531 | 31.80 | 29.52 | 19.20 | 19.46 | 61.33 | 38.66 | 0.037 | -0.006 |
| *P.dumerilianus* | 1538 | 31.92 | 28.47 | 20.54 | 19.05 | 60.40 | 39.59 | 0.057 | 0.037 |
| *P. unifilis* | 1537 | 33.31 | 30.38 | 18.67 | 17.63 | 63.69 | 36.30 | 0.045 | 0.028 |
| **rRNA genes** | | | | | | | | | |
| *P. sylhetensis* | 2560 | 37.53 | 21.44 | 16.95 | 24.06 | 58.98 | 41.01 | 0.272 | -0.173 |
| *B. trivittata* | 2568 | 37.26 | 20.52 | 17.44 | 24.76 | 57.78 | 42.21 | 0.289 | -0.173 |
| *C. amboinensis* | 2572 | 37.67 | 21.38 | 16.95 | 23.98 | 59.05 | 40.94 | 0.275 | -0.171 |
| *C. atripons* | 2561 | 38.73 | 21.98 | 16.40 | 22.88 | 60.71 | 39.28 | 0.275 | -0.165 |
| *H. annandalii* | 2563 | 39.32 | 22.27 | 16.07 | 22.31 | 61.60 | 38.39 | 0.276 | -0.162 |
| *M. caspica* | 2568 | 37.88 | 21.65 | 16.78 | 23.67 | 59.54 | 40.46 | 0.272 | -0.170 |
| *N. platynota* | 2573 | 38.55 | 22.46 | 16.28 | 22.69 | 61.01 | 38.98 | 0.263 | -0.164 |
| *S. bealei* | 2574 | 38.11 | 21.87 | 16.70 | 23.31 | 59.98 | 40.01 | 0.270 | -0.165 |
| *A. cartilaginea* | 2578 | 39.56 | 21.45 | 16.17 | 22.80 | 61.01 | 38.98 | 0.296 | -0.170 |
| *A. ferox* | 2583 | 40.03 | 21.21 | 15.87 | 22.88 | 61.24 | 38.75 | 0.307 | -0.180 |
| *C. indica* | 2569 | 39.35 | 20.39 | 16.54 | 23.70 | 59.75 | 40.24 | 0.317 | -0.177 |
| *D. subplana* | 2576 | 39.09 | 21.73 | 16.14 | 23.02 | 60.83 | 39.16 | 0.285 | -0.175 |
| *L. punctata* | 2546 | 38.41 | 21.95 | 16.92 | 22.70 | 60.36 | 39.63 | 0.272 | -0.145 |
| *N. formosa* | 2592 | 40.23 | 21.83 | 15.58 | 22.33 | 62.07 | 37.92 | 0.296 | -0.178 |
| *P. steindachneri* | 2583 | 39.25 | 21.29 | 16.22 | 23.22 | 60.55 | 39.45 | 0.296 | -0.177 |
| *P. cantorii* | 2566 | 39.63 | 20.18 | 16.40 | 23.77 | 59.82 | 40.17 | 0.325 | -0.183 |
| *P. sinensis* | 2685 | 39.14 | 22.57 | 15.97 | 22.30 | 61.71 | 38.28 | 0.268 | -0.165 |
| *R. swinhoei* | 2572 | 38.60 | 21.34 | 16.75 | 23.28 | 59.95 | 40.04 | 0.287 | -0.163 |
| *T. triunguis* | 2585 | 39.22 | 19.76 | 16.05 | 24.95 | 58.99 | 41.00 | 0.329 | -0.217 |
| *I. elongata* | 2571 | 39.36 | 21.47 | 16.02 | 23.14 | 60.83 | 39.16 | 0.294 | -0.181 |
| *M. tornieri* | 2578 | 38.51 | 21.64 | 16.40 | 23.42 | 60.16 | 39.83 | 0.280 | -0.176 |
| *M. emys* | 2580 | 37.79 | 21.27 | 16.89 | 24.03 | 59.07 | 40.93 | 0.279 | -0.174 |
| *S. pardalis* | 2582 | 38.76 | 20.87 | 16.77 | 23.58 | 59.64 | 40.35 | 0.300 | -0.168 |
| *T. graeca* | 2570 | 38.91 | 22.14 | 16.38 | 22.56 | 61.05 | 38.94 | 0.274 | -0.158 |
| *C. caretta* | 1611 | 39.41 | 23.21 | 16.20 | 21.16 | 62.63 | 37.36 | 0.258 | -0.132 |
| *C. mydas* | 2581 | 38.24 | 21.92 | 16.50 | 23.32 | 60.17 | 39.83 | 0.271 | -0.171 |
| *E. imbricata* | 2580 | 38.56 | 21.82 | 16.27 | 23.33 | 60.38 | 39.61 | 0.277 | -0.178 |
| *L. olivacea* | 2579 | 39.00 | 22.52 | 16.01 | 22.45 | 61.53 | 38.46 | 0.267 | -0.167 |
| *N. depressa* | 2577 | 38.68 | 21.53 | 16.37 | 23.39 | 60.22 | 39.77 | 0.284 | -0.176 |
| *C. serpentina* | 2571 | 37.72 | 23.06 | 16.45 | 22.75 | 60.79 | 39.20 | 0.241 | -0.160 |
| *M. temminckii* | 2570 | 37.85 | 23.58 | 16.38 | 22.17 | 61.44 | 38.56 | 0.232 | -0.150 |
| *C. picta bellii* | 2583 | 39.02 | 21.33 | 16.72 | 22.91 | 60.35 | 39.64 | 0.293 | -0.156 |
| *M. terrapin terrapin* | 2589 | 38.58 | 21.97 | 16.72 | 22.71 | 60.56 | 39.43 | 0.274 | -0.151 |
| *T. scripta* | 2589 | 38.50 | 21.86 | 16.72 | 22.90 | 60.37 | 39.62 | 0.275 | -0.155 |
| *K. leucostomum* | 2557 | 39.26 | 24.16 | 15.99 | 20.57 | 63.43 | 36.56 | 0.237 | -0.125 |
| *S. carinatus* | 2562 | 39.22 | 23.06 | 15.92 | 21.78 | 62.29 | 37.70 | 0.259 | -0.155 |
| *C. insculpta* | 2567 | 39.42 | 21.15 | 16.47 | 22.94 | 60.57 | 39.42 | 0.301 | -0.164 |
| *P.megacephalum* | 2565 | 37.27 | 23.04 | 16.88 | 22.80 | 60.31 | 39.68 | 0.235 | -0.149 |
| *C. expansa* | 2569 | 38.73 | 22.61 | 16.69 | 21.95 | 61.34 | 38.65 | 0.262 | -0.136 |
| *C. fimbriata* | 2566 | 38.73 | 22.17 | 16.83 | 22.25 | 60.91 | 39.08 | 0.271 | -0.138 |
| *E.branderhorsti* | 2567 | 38.95 | 21.73 | 16.47 | 22.82 | 60.69 | 39.30 | 0.283 | -0.161 |
| *E. macrurus* | 2577 | 38.88 | 22.19 | 16.53 | 22.39 | 61.07 | 38.92 | 0.273 | -0.150 |
| *E.subglobosa* | 2575 | 38.21 | 22.79 | 16.62 | 22.36 | 61.01 | 38.99 | 0.252 | -0.147 |
| *M. hogei* | 2575 | 37.90 | 21.98 | 17.35 | 22.75 | 59.88 | 40.11 | 0.265 | -0.134 |
| *M. bellii* | 2579 | 38.23 | 22.33 | 16.55 | 22.87 | 60.56 | 39.43 | 0.262 | -0.160 |
| *P.platycephala* | 2583 | 38.40 | 21.60 | 16.64 | 23.34 | 60.00 | 39.99 | 0.280 | -0.167 |
| *P.umbrina* | 2553 | 38.42 | 24.05 | 16.49 | 21.03 | 62.47 | 37.52 | 0.230 | -0.121 |
| *P. hilarii* | 2572 | 37.09 | 21.77 | 18.16 | 22.98 | 58.86 | 41.14 | 0.260 | -0.117 |
| *P. subrufa* | 2572 | 36.46 | 22.58 | 17.30 | 23.63 | 59.05 | 40.94 | 0.235 | -0.154 |
| *P. castaneus* | 2568 | 36.02 | 23.13 | 17.48 | 23.36 | 59.15 | 40.84 | 0.217 | -0.143 |
| *P.dumerilianus* | 2566 | 37.49 | 20.03 | 17.84 | 24.63 | 57.52 | 42.47 | 0.303 | -0.159 |
| *P. unifilis* | 2555 | 36.43 | 23.40 | 17.26 | 22.89 | 59.84 | 40.15 | 0.217 | -0.140 |
| **Control region** | | | | | | | | | |
| *P. sylhetensis* | 1067 | 31.67 | 33.36 | 13.12 | 21.83 | 65.04 | 34.95 | -0.025 | -0.249 |
| *B. trivittata* | 947 | 31.67 | 33.26 | 12.98 | 22.07 | 64.94 | 35.05 | -0.024 | -0.259 |
| *C. amboinensis* | 1182 | 33.16 | 40.27 | 10.74 | 15.82 | 73.43 | 26.56 | -0.096 | -0.191 |
| *C. atripons* | 981 | 34.76 | 34.76 | 12.13 | 18.34 | 69.52 | 30.47 | 0.000 | -0.204 |
| *H. annandalii* | 1095 | 34.79 | 36.43 | 10.59 | 18.17 | 71.23 | 28.76 | -0.023 | -0.263 |
| *M. caspica* | 1223 | 33.44 | 41.29 | 9.89 | 15.37 | 74.73 | 25.26 | -0.105 | -0.216 |
| *N. platynota* | 1457 | 32.18 | 40.28 | 8.30 | 19.21 | 72.47 | 27.52 | -0.111 | -0.396 |
| *S. bealei* | 1048 | 32.72 | 36.54 | 11.92 | 18.79 | 69.27 | 30.72 | -0.055 | -0.223 |
| *A. ferox* | 1357 | 31.61 | 35.74 | 10.31 | 22.32 | 67.35 | 32.64 | -0.061 | -0.367 |
| *C. indica* | 1227 | 33.00 | 32.92 | 10.10 | 23.96 | 65.93 | 34.06 | 0.001 | -0.406 |
| *D. subplana* | 1820 | 30.54 | 32.91 | 10.60 | 25.93 | 63.46 | 36.53 | -0.037 | -0.419 |
| *L. punctata* | 1008 | 34.92 | 31.74 | 11.80 | 21.52 | 66.66 | 33.33 | 0.047 | -0.291 |
| *N. formosa* | 1604 | 33.97 | 36.34 | 8.54 | 21.13 | 70.32 | 29.67 | -0.033 | -0.424 |
| *P. steindachneri* | 1736 | 29.37 | 36.34 | 13.19 | 21.08 | 65.72 | 34.27 | -0.106 | -0.230 |
| *P. cantorii* | 1994 | 34.65 | 33.75 | 8.62 | 22.96 | 68.40 | 31.59 | 0.013 | -0.454 |
| *P. sinensis* | 1830 | 29.50 | 31.03 | 10.87 | 28.57 | 60.54 | 39.45 | -0.025 | -0.448 |
| *R. swinhoei* | 1471 | 31.67 | 34.46 | 10.12 | 23.72 | 66.14 | 33.85 | -0.042 | -0.401 |
| *T. triunguis* | 1078 | 35.80 | 31.26 | 9.18 | 23.74 | 67.06 | 32.93 | 0.067 | -0.442 |
| *I. elongata* | 1290 | 33.41 | 35.96 | 10.69 | 19.92 | 69.38 | 30.62 | -0.036 | -0.301 |
| *M. tornieri* | 1683 | 35.17 | 34.40 | 8.49 | 21.92 | 69.57 | 30.42 | 0.011 | -0.441 |
| *M. emys* | 948 | 34.07 | 34.28 | 11.39 | 20.25 | 68.35 | 31.64 | -0.003 | -0.280 |
| *S. pardalis* | 3885 | 20.97 | 32.66 | 21.15 | 25.19 | 53.64 | 46.35 | -0.217 | -0.087 |
| *T. graeca* | 3771 | 30.52 | 40.38 | 8.19 | 20.89 | 70.91 | 29.09 | -0.139 | -0.436 |
| *C. caretta* | 1134 | 35.53 | 36.59 | 10.40 | 17.46 | 72.13 | 27.86 | -0.014 | -0.253 |
| *C. mydas* | 982 | 35.84 | 31.36 | 12.01 | 20.77 | 67.21 | 32.79 | 0.066 | -0.267 |
| *E. imbricata* | 974 | 35.62 | 32.75 | 11.91 | 19.71 | 68.37 | 31.62 | 0.042 | -0.246 |
| *L. olivacea* | 600 | 32.33 | 35.16 | 14.00 | 18.50 | 67.50 | 32.50 | -0.042 | -0.138 |
| *N. depressa* | 760 | 32.63 | 32.89 | 13.94 | 20.52 | 65.52 | 34.47 | -0.004 | -0.190 |
| *C. serpentina* | 1125 | 32.35 | 37.06 | 12.00 | 18.57 | 69.42 | 30.57 | -0.067 | -0.215 |
| *M. temminckii* | 1063 | 33.67 | 35.27 | 11.75 | 19.28 | 68.95 | 31.04 | -0.023 | -0.242 |
| *C. picta bellii* | 1034 | 32.78 | 34.33 | 13.05 | 19.82 | 67.11 | 32.88 | -0.023 | -0.205 |
| *M. terrapin terrapin* | 1036 | 32.14 | 34.84 | 13.32 | 19.69 | 66.98 | 33.01 | -0.040 | -0.193 |
| *T. scripta* | 1120 | 32.14 | 34.46 | 12.94 | 20.44 | 66.60 | 33.39 | -0.034 | -0.224 |
| *K. leucostomum* | 1006 | 33.79 | 32.80 | 10.93 | 22.46 | 66.60 | 33.40 | 0.014 | -0.345 |
| *S. carinatus* | 1003 | 33.00 | 33.20 | 11.26 | 22.53 | 66.20 | 33.79 | -0.003 | -0.333 |
| *C. insculpta* | 972 | 37.24 | 27.46 | 10.28 | 25.00 | 64.71 | 35.28 | 0.151 | -0.416 |
| *P.megacephalum* | 1139 | 35.11 | 30.55 | 10.97 | 23.35 | 65.67 | 34.32 | 0.069 | -0.360 |
| *C. expansa* | 941 | 33.26 | 33.58 | 14.13 | 19.02 | 66.84 | 33.15 | -0.004 | -0.147 |
| *C. fimbriata* | 1218 | 37.60 | 31.52 | 10.75 | 20.11 | 69.13 | 30.87 | 0.087 | -0.303 |
| *E.branderhorsti* | 892 | 34.08 | 32.17 | 11.88 | 21.86 | 66.25 | 33.74 | 0.028 | -0.295 |
| *E. macrurus* | 918 | 32.57 | 34.74 | 11.43 | 21.24 | 67.32 | 32.68 | -0.032 | -0.300 |
| *E.subglobosa* | 882 | 32.99 | 35.37 | 12.35 | 19.27 | 68.36 | 31.63 | -0.034 | -0.218 |
| *M. hogei* | 953 | 31.05 | 34.20 | 14.06 | 20.67 | 65.26 | 34.73 | -0.048 | -0.190 |
| *M. bellii* | 888 | 33.55 | 34.45 | 12.27 | 19.70 | 68.01 | 31.98 | -0.013 | -0.232 |
| *P.platycephala* | 984 | 30.89 | 33.33 | 15.44 | 20.32 | 64.22 | 35.77 | -0.038 | -0.136 |
| *P.umbrina* | 863 | 31.17 | 36.26 | 13.90 | 18.65 | 67.43 | 32.56 | -0.075 | -0.145 |
| *P. hilarii* | - | - | - | - | - | - | - | - | - |
| *P. subrufa* | 1194 | 37.85 | 34.84 | 8.62 | 18.67 | 72.69 | 27.30 | 0.041 | -0.368 |
| *P. castaneus* | 1180 | 31.18 | 35.50 | 10.42 | 22.88 | 66.69 | 33.30 | -0.064 | -0.374 |
| *P.dumerilianus* | 985 | 32.28 | 26.70 | 15.93 | 25.07 | 58.98 | 41.01 | 0.094 | -0.222 |
| *P. unifilis* | 985 | 28.93 | 32.38 | 12.69 | 25.99 | 61.32 | 38.68 | -0.056 | -0.343 |
