# Supplementary material for: The complete mitochondrial genome of the endangered Assam Roofed Turtle, Pangshura sylhetensis (Testudines: Geoemydidae): Genomic features and phylogeny
Source: PLoS One. 2020 Apr 23;15(4):e0225233. doi: 10.1371/journal.pone.0225233 (PMC7179895; doi:10.1371/journal.pone.0225233)
Supplement: S5 Table — (DOC) [file pone.0225233.s011.doc]

**Table S5** Frequency of start and stop codon distribution within the complete mitogenomes of 52 Testudines.

| **Genes** | **Start Codons** | **Nos. Species** | **Percentage** | **Stop Codons** | **Nos. Species** | **Percentage** |
| --- | --- | --- | --- | --- | --- | --- |
| *nad1* | ATG | 33 | 64.71 | TAA | 6 | 11.76 |
|  | GTG | 1 | 1.96 | TAG | 15 | 29.41 |
|  | ATA | 10 | 19.61 | T | 3 | 5.88 |
|  | ATT | 8 | 15.69 | TA | 28 | 54.90 |
| *nad2* | ATG | 42 | 82.35 | TAA | 10 | 19.61 |
|  | ATA | 6 | 11.76 | TAG | 12 | 23.53 |
|  | ATT | 1 | 1.96 | AGA | 2 | 3.92 |
|  | ATC | 3 | 5.88 | TA | 3 | 5.88 |
|  |  |  |  | T | 25 | 49.02 |
| *cox1* | ATG | 14 | 27.45 | TAA | 12 | 23.53 |
|  | GTG | 37 | 72.55 | TAG | 2 | 3.92 |
|  | ATT | 1 | 1.96 | AGA | 11 | 21.57 |
|  |  |  |  | T | 2 | 3.92 |
|  |  |  |  | AGG | 25 | 49.02 |
| *cox2* | ATG | 44 | 86.27 | TAA | 35 | 68.63 |
|  | GTG | 2 | 3.92 | TAG | 5 | 9.80 |
|  | ATA | 6 | 11.76 | T | 11 | 21.57 |
|  |  |  |  | TA | 1 | 1.96 |
| *atp8* | ATG | 49 | 96.08 | TAA | 48 | 94.12 |
|  | ATA | 3 | 5.88 | TAG | 4 | 7.84 |
| *atp6* | ATG | 48 | 94.12 | TAA | 42 | 82.35 |
|  | ATA | 3 | 5.88 | TAG | 5 | 9.80 |
|  | GTG | 1 | 1.96 | TA | 4 | 7.84 |
|  |  |  |  | T | 1 | 1.96 |
| *cox3* | ATG | 42 | 82.35 | TAA | 2 | 3.92 |
|  | ATA | 10 | 19.61 | TA | 6 | 11.76 |
|  |  |  |  | T | 44 | 86.27 |
| *nad3* | ATG | 34 | 66.67 | TAA | 8 | 15.69 |
|  | ATA | 13 | 25.49 | TAG | 5 | 9.80 |
|  | ATT | 2 | 3.92 | AGA | 5 | 9.80 |
|  | ATC | 3 | 5.88 | T | 33 | 64.71 |
|  |  |  |  | TA | 1 | 1.96 |
| *nad4l* | ATG | 37 | 72.55 | TAA | 50 | 98.04 |
|  | ATA | 10 | 19.61 | TA | 1 | 1.96 |
|  | ATT | 2 | 3.92 | T | 1 | 1.96 |
|  | ATC | 3 | 5.88 |  |  |  |
| *nad4* | ATG | 45 | 88.24 | TAA | 11 | 21.57 |
|  | GTG | 5 | 9.80 | TAG | 2 | 3.92 |
|  | ATA | 1 | 1.96 | AGA | 2 | 3.92 |
|  | ATC | 1 | 1.96 | T | 36 | 70.59 |
|  |  |  |  | A | 1 | 1.96 |
| *nad5* | ATG | 49 | 96.08 | TAA | 48 | 94.12 |
|  | GTG | 1 | 1.96 | TAG | 3 | 5.88 |
|  | ATA | 1 | 1.96 | A | 1 | 1.96 |
|  | ATT | 1 | 1.96 |  |  |  |
| *nad6* | ATG | 46 | 90.20 | TAA | 2 | 3.92 |
|  | ATA | 6 | 11.76 | TAG | 7 | 13.73 |
|  |  |  |  | AGA | 15 | 29.41 |
|  |  |  |  | AGG | 28 | 54.90 |
| *cytb* | ATG | 38 | 74.51 | TAA | 22 | 43.14 |
|  | ATA | 11 | 21.57 | TAG | 1 | 1.96 |
|  | ATT | 1 | 1.96 | TA | 4 | 7.84 |
|  | ATC | 2 | 3.92 | T | 23 | 45.10 |
|  |  |  |  | A | 2 | 3.92 |
